# Palaeoproteomics identifies beaver fur in Danish high-status Viking Age burials - direct evidence of fur trade

**DOI:** 10.1371/journal.pone.0270040

**Published:** 2022-07-27

**Authors:** Luise Ørsted Brandt, Alberto J. Taurozzi, Meaghan Mackie, Mikkel-Holger S. Sinding, Filipe Garrett Vieira, Anne Lisbeth Schmidt, Charlotte Rimstad, Matthew J. Collins, Ulla Mannering

**Affiliations:** 1 The GLOBE Institute, University of Copenhagen, København K, Denmark; 2 The Novo Nordisk Foundation Center for Protein Research, University of Copenhagen, København K, Denmark; 3 Department of Biology, University of Copenhagen, København K, Denmark; 4 The National Museum of Denmark, Copenhagen, Denmark; New York State Museum, UNITED STATES

## Abstract

Fur is known from contemporary written sources to have been a key commodity in the Viking Age. Nevertheless, the fur trade has been notoriously difficult to study archaeologically as fur rarely survives in the archaeological record. In Denmark, fur finds are rare and fur in clothing has been limited to a few reports and not recorded systematically. We were therefore given access to fur from six Danish high status graves dated to the Viking Age. The fur was analysed by aDNA and palaeoproteomics methods to identify the species of origin in order to explore the Viking Age fur trade. Endogenous aDNA was not recovered, but fur proteins (keratins) were analysed by MALDI-TOF-MS and LC-MS/MS. We show that Viking Age skin clothing were often composites of several species, showing highly developed manufacturing and material knowledge. For example, fur was produced from wild animals while leather was made of domesticates. Several examples of beaver fur were identified, a species which is not native to Denmark, and therefore indicative of trade. We argue that beaver fur was a luxury commodity, limited to the elite and worn as an easily recognisable indicator of social status.

## Introduction

**Fig 1:**
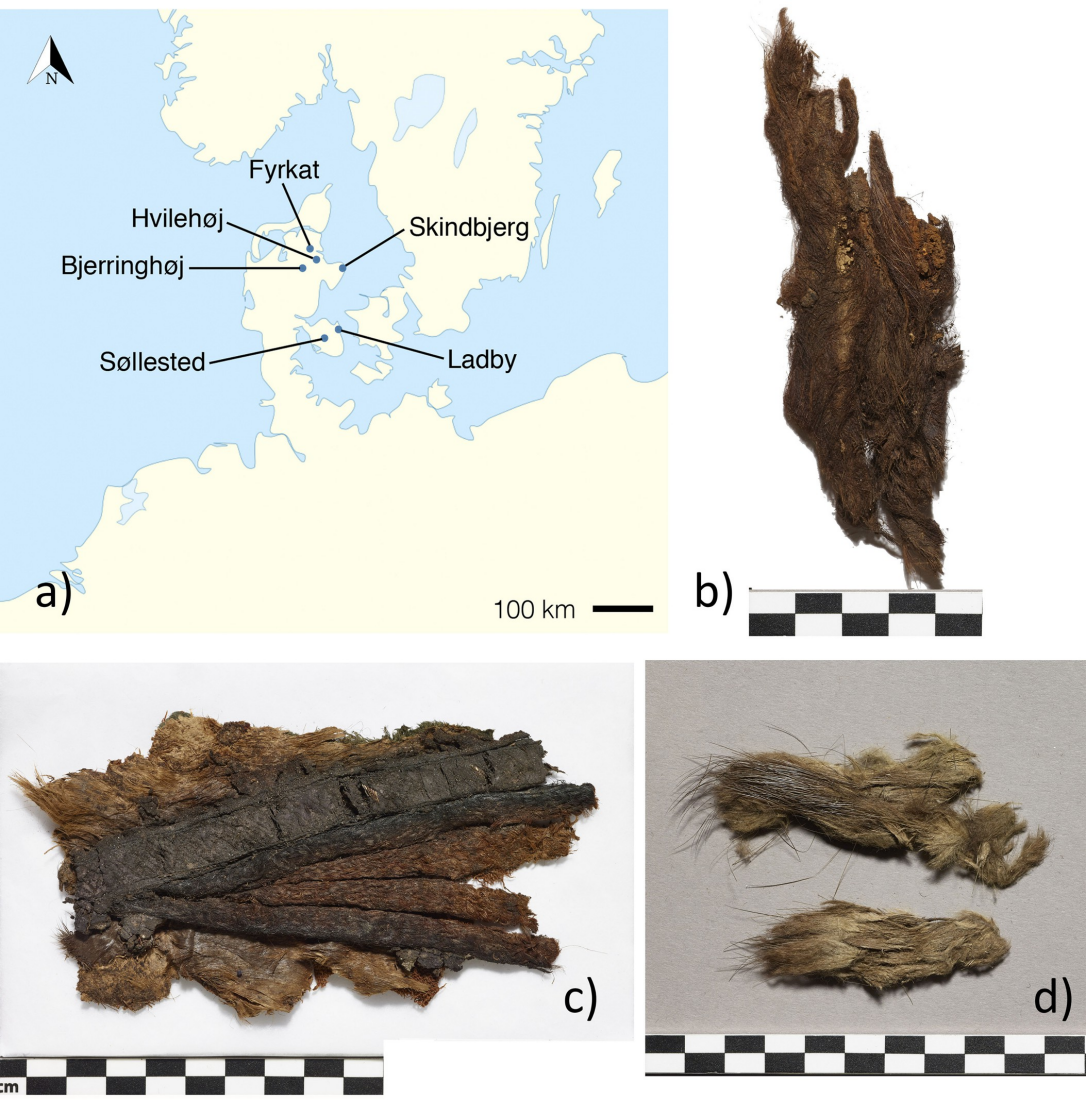
Map of studied sites (a) and examples of included fur: b) Hvilehøj C4273-97, fragment 1, c) Hvilehøj C4280c, d) Bjerringhøj C143. Graphics: Luise Ørsted Brandt and Charlotte Rimstad. Photos: Roberto Fortuna, National Museum of Denmark.

One of the major characteristics of the Viking Age, the final era of the Scandinavian Late Iron Age spanning from around 800 to around 1050 CE, is extensive international trade and exchange of goods [1, 2]. Contemporary written sources describe how fur from wild animals hunted in current day Northern Scandinavia and Russia like fox, beaver, marten, ermine and sable were amongst the pivotal commodities that were brought via the eastern trade routes to the growing Arab fur market in exchange for beads, silver, gold and silk [3, 4]. An example of the economic value of imported fur is given by the Arab traveller, geographer, and historian al-Mas´ūdī from Baghdad who in 943 wrote:”*The black furs are worn by Arab and non-Arab kings…* …*They make hats*, *caftans and fur coats out of them*. *There is no king who does not possess a fur coat or a caftan lined with the black fox fur of the Burtās*” [5, 6]. While the significance of imported fur for the Arab market is well-described, its use and value as a visual marker of status in Scandinavia is less well understood. Fur procurement has been notoriously difficult to study in an archaeological context as fur’s organic nature leads to its rapid degradation. In addition, its presence and appearance has not been systematically recorded. In the Viking Age market place Birka in Sweden, fur has been preserved and recorded in connection with penannular brooches in burials, showing that fur was part of the clothing [7]. In Denmark, the textile catalogue by Bender Jørgensen [8] mentions a few examples of preserved fur in Viking Age burials, but no systematic examination has been performed and, until recently, only a few minor studies have been published [9, 10]. A wide variety of burial customs were present in Viking Age Denmark, from cremations to inhumation graves with both social, regional and chronological differences related to the introduction of Christianity [11]. There is bias in the survival of fur; the few acknowledged examples of Viking Age fur clothing that have survived are from elaborate burials belonging to the elite. For instance, fur was found in waggon bed burials (Hvilehøj, Fyrkat), graves with wooden constructions (Bjerringhøj, Skindbjerg, Søllested) and in a ship burial (Ladby) (Fig 1). Most of these burials were also covered by large mounds. The complex grave constructions, as well as contact with metal grave goods, have in some cases aided the preservation of organic materials such as fur from clothing, accessories or grave furnishing [12].

If preserved, one of the first serious obstacles in fur studies is the species identification of the hairs which often requires specialized knowledge and/or access to advanced analyses and equipment [13].

### Analytical strategies

In cases where Viking Age fur has survived, species have been almost exclusively identified through morphological characteristics of the hairs through transmitted light microscopy. In 1933, fibres from some of the Birka graves were identified as squirrel, marten, beaver and possibly bear [14]. Later, as technologies advanced, fibres from other graves have been identified as sheep wool and beaver fur with Scanning Electron Microscopy (SEM) [7]. However, the reproducibility of microscopy relies on extended knowledge and experience to account for intra species variation, as well as suitable reference collections, and is complicated further by diagenesis [15]. As an example of the difficulties of species identification by microscopy, fur from the Bjerringhøj grave in Denmark was first proposed to be beaver or marten [16], then was assigned as marmot after another independent analysis [17]. Similarly, fur from the Danish Hvilehøj grave was also at first described as beaver [18], but later identified as marmot [10, 19]. Neither marmot nor beaver would have been local to Denmark in the Viking Age [20] and thus indicate a trade of fur, but would have come by different trade routes, from the south and east or the north, respectively, and therefore accurate identification is still important to the interpretation of the activities and connections of the sites.

To counteract difficulties associated with species IDs based solely on hair morphology, developments of ancient DNA (aDNA) technologies over the last decades have led to successful investigations of archaeological hair, wool and fur materials [21–24]. In addition, zooarchaeology by mass spectrometry (ZooMS) is a commonly employed technique for species ID from collagen containing samples such as skin and bone. ZooMS utilises matrix-assisted laser desorption/ionization time-of-flight (MALDI-TOF) mass spectrometry to generate species specific peptide mass fingerprints from trypsin digested collagen. A similar approach can be used to provide species ID from keratinaceous samples such as nail, hoof, horn, beak, feathers, skin, wool and fur. Analysis of keratins in archaeological textiles made from such materials has, therefore, already provided successful species identifications at many sites [25–31]. The potential of archaeological hair samples was demonstrated on archaeological pelt and textiles found in connection to copper-alloy artefacts [30] and the clothing of the Neolithic Tyrolean Iceman Ötzi [27, 28]. Validation of PMF species ID markers can be performed by liquid chromatography coupled to tandem mass spectrometry (LC-MS/MS). Through this technique, the amino acid sequence of the PMF peptides is determined, allowing robust confirmation of phylogenetically informative amino acid substitutions. Whilst LC-MS/MS analysis is the ‘gold standard’ of proteomics, it is low-throughput in comparison to PMF methods which are the mainstay of screening and large scale studies. When comparing these varied bioarchaeology approaches currently available, perhaps the most significant advantage of aDNA vs protein analyses (especially those that rely on PMF), is species resolution. Genomic DNA sequencing allows access to many sites of variability, particularly intronic regions allowing unparalleled species resolution and the ability to reveal structure in ancient populations and map ancient individuals to geographical regions. The latter was recently demonstrated for Atlantic walruses (27). In contrast, PMF targets only a few highly abundant proteins. For instance, keratin proteins are the product of a few genes, therefore PMF of keratin only allows access to the variation within the exons of the keratin genes. However, DNA has a significant disadvantage: namely its poor preservation in many archaeological environments [24]. In contrast, proteins are inherently more resistant to degradation and may be identifiable in samples that no longer contain amplifiable endogenous DNA [15]. To summarize, if endogenous aDNA is recoverable it provides a superior approach for species identification and phylogenetic analyses. On the other hand, if aDNA is not recoverable/not present, protein analysis allows a robust, albeit lower resolution, approach that remains sufficient for most applications. This article utilizes several biomolecular methods (aDNA, PMF and LC-MS/MS) as well as microscopy (see S2 Table in S1 File) to analyze fur samples from the most extraordinary Viking Age graves from modern-day Denmark (see S1 Table in S1 File) to establish the material use of fur in the Viking Age, shed light on Viking Age fur trade, and examine fur as a visual identifier of elite status.

## Materials and methods

The fur items examined here derive from six richly equipped burials belonging to the very top of society in 10th century Denmark (Fig 1 and S1 Table in S1 File). Based on the archaeological material, the graves contained respectively three women, two men and one of unknown biological sex. Prior to destructive biomolecular analysis, we performed species screening by transmitted light microscopy of hair (See methodology in S1 File and Table 1). Then the selected items were sampled for fur. Fur samples were analysed by ancient DNA using a next generation sequencing shotgun approach (see methodology in S1 File). Subsequently, the sample material was subjected to the analysis of peptide mass fingerprints (PMF) of keratins using MALDI-TOF mass spectrometry and six samples were also verified by liquid chromatography–tandem mass spectrometry (LC-MS/MS). The samples were prepared based on a previous protocol [30 and see further methodology in S1 File].

**Table 1:** Identifications of samples based on DNA, PMF, LC-MS/MS, and hair microscopy.

Site	Inventory no.†	DNA	PMF	LC-MS/MS	Microscopy
Hvilehøj	C4273-97, fragment 19	No ID	*Castor*	*Castor* (*canadensis*)*	Not analysed
	C4273-97, fragment 1	No ID	Bovidae/cervidae	*Ovis aries*	*Equus*?
	C4273-97, fragment 60	No ID	Closest match to *Castor*	Sciuridae (probably *Sciurus vulgaris*)	*Bos taurus*?
	AdC4291, fragment 12	No ID	*Castor*	Not analysed	*Bos taurus*?
	C4280c	No ID	Closest match to *Castor*	Not analysed	*Bos taurus*?
	C4280b, fragment 11	No ID	*Castor*	Not analysed	*Castor*
Bjerringhøj	C150, fragment 3	No ID	Bovidae/cervidae	Not analysed	*Ovis aries/Equus*?
	AdC143	No ID	*Castor*	*Castor* (*canadensis*)*	*Canidae*
	C143	No ID	*Castor*	Not analysed	*Castor*
Skindbjerg	C13324	No ID	No ID	Not analysed	No ID
Søllested	25595	No ID	No ID‡	Not analysed	*Canidae*?
Ladby	C30238, L4 504, internal no. A	No ID	Bovidae/cervidae?	Not analysed	*Ovis aries*?
Ladby	C30238, L4 504, internal no. B	No ID	No ID	Not analysed	*Canidae*?
Fyrkat	Grave 4, D158-1966, GuHCl protocol	Not analysed	Mustelidae/Ursidae	*Mustela* sp.	Not analysed
Fyrkat	Grave 4, D158-1966, Urea protocol	Not analysed	Mustelidae/Ursidae	Mustelidae (probably *Mustela* sp.)	Not analysed

† Hvilehøj and Bjerringhøj numbers are listed according to a forthcoming catalogue of the finds [34].

* European beaver (*Castor fiber*) sequences are not publically available and not present in the reference database. Therefore, the samples were assigned to North American beaver (*Castor canadensis*).

‡ Another subsample of the same object was identified as cattle by ZooMS (Zooarchaeology by Mass Spectrometry) in [35]. Therefore this sample can also be assigned to cattle.

## Results

### Results of microscopy

While the results of PMF and LC-MS/MS are in agreement in all six cases, microscopy agrees with PMF in four of 12 possible cases. In three cases, PMF failed to provide an identification and the correspondence cannot be evaluated, while in five cases, microscopy disagrees with PMF or PMF and LC-MS/MS (Table 1).

### Results of DNA

Of the fifteen samples included in this study, thirteen samples were selected for DNA sequencing analysis. All thirteen samples were positively amplified during PCR and all blanks failed in amplification, excluding contamination of reagents and cross contamination. These samples were then successfully sequenced, resulting in between 1,014,051 and 7,351,856 trimmed reads per sample (S3 Table in S1 File). The number of reads mapping to any reference was extremely low, with the highest fraction of unique reads mapping (see S3 Table in S1 File) ranging from 0.004% (44 reads) to 0.00005% (1 read). These mapping results testify to poor endogenous DNA preservation in the samples, and no reliable DNA analysis or interpretation can be made.

### Results of PMF of keratin

**Fig 2:**
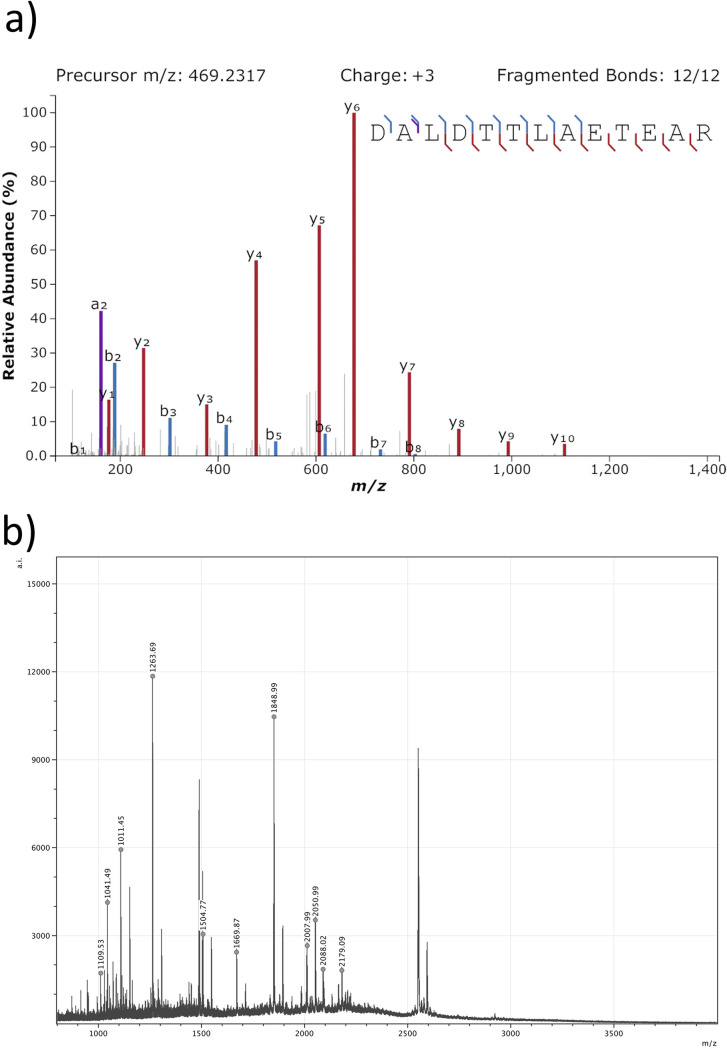
Panel A: MS/MS spectrum of the peptide DALDTTLAETEAR from Hvilehøj C4273-97, fragment 19 which is specific to *Castor (canadensis*) keratin, type I cuticular Ha5. The spectrum was produced using Interactive Peptide Spectral Annotator [33]. Panel B: PMF spectrum from Hvilehøj C4273-97, fragment 19 which is specific to beaver based on the peaks 1669, 2050, and 2179.

Fourteen different items of fur from the six sites were submitted to PMF analysis. The fur sample from Fyrkat had two different extraction methods attempted, resulting in 15 analyses in total. Of the 15, 12 gave some indication of species identification (Table 1). Unfortunately, the samples from Skindbjerg, Søllested, and one from Ladby all were unable to provide meaningful results. Of the successful samples, five samples were identified as beaver based on the presence of the peak *m/z* 1669, which is unique to this species [31]. The identifications were all supported by additional peaks *m/z* 2050 and 2179, currently only observed in beaver as well as peaks *m/z* 2088 and 2163 limiting the identification to rodents, opossum or carnivores (S5 Table in S1 File and Fig 2). In addition, two samples (Hvilehøj C4273-97, fragment 60 and Hvilehøj C4280c) have their closest match to beaver, but may be other closely related species based on the peak *m/z* 1518 which is currently only recorded in muskrat and a minor *m/z* 1669 in Hvilehøj C4280c. Currently only a few species within Rodentia are listed as references, why other closely related species could also be possible candidates for these samples. Hvilehøj C4273-97, fragment 1 and Bjerringhøj C150, fragment 3 could be assigned to the families of either bovids or cervids based on the peak *m/z* 1834 (S5 Table in S1 File). One sample from Ladby (C30238, L4 504, A) is also tentatively assigned to bovids or cervids, but the peak *m/z* 1669 confuses this identification as this is unique to beaver. The two extractions from Fyrkat were assigned to either Mustelidae or Ursidae based on the peaks *m/z* 2035? and 2164 in combination with *m/z* 2088. Several peaks had clear signs of deamidation indicating that the markers are more likely to be genuine and not contamination [see S1 File and 32]. A few peaks that may be human contamination can be observed. This is unsurprising as many of the samples have been handled for more than a century (See S5 Table in S1 File).

### Results of LC-MS/MS

The five samples (six analyses with the two Fyrkat extractions) examined by LC-MS/MS either confirmed or narrowed down the identifications by PMF (Table 1). Two samples (Hvilehøj C4273-97, fragment 19 and Bjerringhøj AdC143) were confirmed as very likely to be beaver. Hvilehøj C4273-97, fragment 60, with a closest match to beaver in PMF, was actually identified to the family Sciuridae, with the most likely assignment as *Sciurus vulgaris* (red squirrel). Hvilehøj C4273-97, fragment 1 was narrowed down from the PMF identification of Bovid/cervid to originating from sheep. The two extractions of the fur from Fyrkat suggest that it derives from the genus *Mustela* (assigned to Mustelidae or Ursidae by PMF). However, it should be noted that these identifications are based on current, and somewhat limited, databases (see Discussion).

## Discussion

Amplification of aDNA was chosen for a large part of the samples because of its potential high resolution, and because previous studies have demonstrated hair as an excellent substrate for aDNA preservation [22, 23, 36, 37], even in one of the contexts sampled for this study [24]. Despite this, none of the analysed samples yielded endogenous aDNA, demonstrating a high degradation of endogenous DNA in the samples. Many factors involved in the preservation of aDNA are still poorly understood, but the level and character of DNA damage is probably largely determined by age and by the environment [38]. Likewise, treatment processes from turning the raw pelts into fur, including pickling, tanning and dyeing, may also explain the advanced aDNA degradation [24, 39, 40].

Keratin showed to be better preserved than aDNA, as seen in the much higher success rate in identifying these samples (12/15 extractions). Though the resolution of PMF is lower than for aDNA, it often allows identification to at least genus level. The five samples re-analysed by LC-MS/MS were also all successful, in some cases to species level. This serves as an important example of the limitations that often occur when working with archaeological samples and how the use of multiple methods can supplement each other.

In addition, the morphological analyses for species identification performed by microscopy (S7 Table and S18 Fig in S1 File) were compared to the biomolecular identifications. Given the known limitations of these methodologies due to obfuscation caused by decay, it is unsurprising that disagreements in species ID were common (~42% of the samples). Both protein based methods were in very close agreement, and therefore a greater weight was given to those identifications as being the most accurate and least susceptible to misinterpretation. A particular example was Bjerringhøj AdC143 which PMF and LC-MS/MS both identified as beaver while microscopy identified it as Canidae. Although improvements to publicly available protein databases are needed (see next paragraph), proteomics was demonstrated to be a superior and objective approach for archaeological samples with high degradation of endogenous DNA compared to microscopy and aDNA analyses and our results may guide future studies of archaeological fibres.

### Current limitations of protein databases for wild fauna

Unfortunately, current protein databases are heavily biased towards model organisms, and are nowhere close to containing all proteins of all species. Therefore, publicly available databases are not complete with regards to species that could potentially be found in the Viking Age. This is the case both for PMF and LC-MS/MS analyses. The current lack of data is critical, as it limits the ability for complete confidence in the taxonomic identification of ancient samples, as species not in the databases cannot be completely ruled out. This is especially important for the LC-MS/MS analyses, as it often has the high resolution required to get to species level, which may look like those identifications are particularly solid.

For example, from the Sciuridae keratin sequences available in public databases, Hvilehøj C427397, fragment 60 was originally identified as most likely an ‘exotic’ alpine marmot (*Marmota marmota marmota*). However, upon closer examination, there were several species not present in the database, including red squirrel (*Sciurus vulgaris*), arguably a more likely Sciuridae identification due to its presence in the local environment [20] and records of squirrel fur objects in the Viking Age [5, 6]. Additionally, a few peptide sequences recovered differed from the available *Marmota* sequences. After the initial searches, it was fortunate that some keratin sequences from the grey squirrel (*Sciurus carolinensis*, native to North America) became available which were added to the search database to represent the *Sciurus* genus. In addition, we were able to search the *S*. *vulgaris* translated nucleotide database on NCBI for keratin proteins discovered with the original Sciuridae search, allowing for sequence fragments of potential red squirrel keratins to also be searched. While these fragments did not cover the entire protein sequences, it allowed for some recovery of peptides not represented in the publicly available sequences. With the new database, we were able to assign new and previously less species-specific proteins to *S*. *carolinensis*, and also identify sequence variants that could come from *S*. *vulgaris* instead (Dataset SI). Therefore, Hvilehøj C427397, fragment 60 has been assigned to Sciuridae, with the most likely species as the local red squirrel instead of the alpine marmot, which greatly changes the interpretation of the object in respect to trade of the raw materials. This highlights considerations of database limitations in the interpretation of LC-MS/MS results for ancient fur samples.

Similar problems occured with the beaver samples, as European beaver (*Castor fiber*) keratins are not represented in the public databases. Therefore, the North American species (*C*. *canadensis*) was used as a surrogate. These two animals are the same genus, but still may have slightly different protein sequences which make identification less certain. Unfortunately, the nucleotide searching method could not be repeated, as no Eurasian beaver genome is currently available and, therefore, beaver assignments were based on *C*. *canadensis* alone.

The identifications are to the best of our ability at the current time, based on currently available knowledge. Therefore, we encourage further research and database generation in this area, both for protein sequence databases and for peptide mass fingerprints. More accurate identifications will allow better interpretations of past production and trade activities, and therefore a greater understanding of people in the past.

### Identified species in Danish Viking age fur

The 15 samples analysed from six different graves showed the presence of fur from wild animals: beaver, squirrel and a mustelid. Amongst these, the furs from Bjerringhøj (C143) and Hvilehøj [18] previously identified to marmot by microscopy, are now identified as beavers. A domesticated species, sheep, was also identified in the Hvilehøj grave in addition to previously reported goatskin in the same grave and cattle skin in the Søllested grave [35].

To discuss the use and purpose of animal species for fur and skin we integrate a few previously identified objects from the same contexts and an additional and contemporary site, Yholm [35]. Based on the original purpose of these items we suggest that they can be divided into three categories: clothing, accessories (including shoes and bags), and grave furnishing. Several of the tested items from Hvilehøj can be clearly identified as clothing, and fragments of several items from Bjerringhøj and Fyrkat are most likely also related to clothing. The shoes from Hvilehøj and Skindbjerg, as well as a purse from Yholm, fall within the category of accessories. Also a fragment from Hvilehøj (E) with a clear seam has been classified as an accessory. Finally, three rolls of fibres from respectively Hvilehøj, Bjerringhøj and Ladby have been identified as deriving from the furnishing of the burials, possibly used as caulking materials for the wooden structures. A few pieces of rolled-up skin might also belong to unidentified fragments of the interior decoration of the burials. The classification of the objects in this study and the previously reported objects are summarised in Table 2.

**Table 2:** Suggested categories of skin and fur and the animal resources they derive from.

Site	Museum no.	Clothing/probably clothing	Shoes, bag, other	Grave furnishing	Identification
Hvilehøj	C4273-97, fragment 19	x			*Castor* (*canadensis*)*
	C4273-97, fragment 1			x	*Ovis aries*
	C4273-97, fragment 60	x			*Sciuridae (probably Sciurus vulgaris)*
	AdC4291 fragment 12	x			*Castor*
	C4273-97, E		x		*Bos taurus*
	C4273-97, F			x?	*Equus*
	C4280c	x			Closest match to *Castor*
	C4280b, fragment 11	x			*Castor*
	C4281, A		x		Capra hircus
	C4281, B		x		*Capra hircus/Rangifer tarandus*
	C4281, C		x		*Capra hircus/Rangifer tarandus*
Bjerringhøj	C150, fragment 3			x	Bovidae/cervidae
	AdC143	x?			*Castor*
	C143	x?			*Castor*
	C142		x		Bovidae/cervidae
Skindbjerg	C13324			x?	No ID
	C13323, A		x		*Capra hircus/Rangifer tarandus*
Søllested‡	25595, fur			x?	No ID
	25595, skin			x?	*Bos taurus*
Yholm	13608		x		Bovidae/cervidae
Ladby	C30238, L4 504, internal no. A			x	Bovidae/cervidae?
Ladby	C30238, L4 504, internal no. B			x?	No ID
Fyrkat	Grave 4, D158-1966, GuHCl protocol	x?			*Mustela* sp.
Fyrkat	Grave 4, D158-1966, Urea protocol	x?			Mustelidae (probably *Mustela* sp.)

Samples marked in blue are reported in [35]. Purple bars mark samples which derive from wild animals, while orange bars mark samples from domesticated animals.

* European beaver (*Castor fiber*) sequences are not publically available and therefore were not present in the reference database. Therefore, the samples were assigned to North American beaver (*Castor canadensis*).

‡ Fur and skin were sampled from the same fragment. Therefore, although the sample of fur was inconclusive, we can conclude that it is also cattle.

As seen from Table 2, the animal resources of fur and skin used for the three suggested categories have a standardised pattern. Fur clothing is exclusively made by fur from wild animals while the accessories and furnishing materials are exclusively made from skin or fibres from domesticated animals. Except for the wool rolls possibly used as caulking and the skin fragments from Ladby, these are all non-hair items. The two materials; leather and fur, thus seem to have been used for different purposes. Fur was a limited, expensive, and in some cases an imported resource, which was only accessible to the few. Therefore it makes sense that it has only been found in clothing where its visual properties could be displayed. The material was also too precious to dehair and turn into leather where its beautiful appearance and exclusiveness could not be admired. In clothing, fur would have acted as an example of conspicuous consumption [41] i.e. as a recognisable luxury product and visible evidence of the high status, which would differentiate the wearer socially and economically. For leather, domesticated animals were common and local and made up an easily accessible resource. The skins of cattle, sheep and goat were moreover well suited for leather objects based on their properties [42] and easier to replace when worn out. For the purpose of caulking, a material known from textile production, sheeps wool, would have been well suited for insulation and sealing of surfaces. Fur therefore seems to have been mainly used in cases where it could be displayed, while leather was used for more everyday objects.

### Local or imported resources?

Domesticated animals such as sheep and cattle were abundant in Viking Age Denmark and would probably have come from the local area. Several species within the genus of *Mustela* as well as red squirrel were also present in Denmark during Viking Age [20] and, therefore, it is possible that the fur identified in the Fyrkat grave and in Hvilehøj (C4273-97, fragment 60) came from locally hunted, but still attractive, fur animals. However, the identification of beaver tells another story. Based on the current Danish animal bone assemblages, the European beaver (*Castor fiber*) had gone extinct in the Danish area already in the Early Bronze Age [20]. Only one later find of beaver dated to the Late Iron or Viking Age comes from the settlement Mysselhøjgård in Lejre, Denmark [43]. The humerus found here does not immediately support imported beaver fur as this bone would have been removed from the fur during the skinning process, but even if small local populations of beavers were present in Denmark, they would not have been able to support a production of fur garments which would require many animals for one single garment. Therefore, beaver skins are expected to be imported in the Viking Age.

### Viking Age fur trade

As previously noted, several contemporary written sources describe the Viking Age fur trade, including the trade routes, the traders, and the specific species of fur traded. According to these sources, fur came from a variety of species including mustelids (such as sable, marten and ermine) squirrel, fox, wolf, beaver, and hare, in addition to skins from domesticated species such as sheep, goat and cattle [3, 5, 6]. Rus´, or Scandinavian Vikings who settled in eastern Europe [44], are described as central stakeholders in the fur trade in Arab sources from the 10th century CE. In the 9th and 10th centuries, the Rus´ brought their fur to the center for fur trade, Bulgar, located on the Volga, from where fur was distributed to the Arab world, Central Asia and Northern Africa [6]. Ibn Hawqal described this trade in 965: “*The honey*, *wax*, *and furs exported from their country come from the territories of the Rūs and the Bulghār*. *This is also the case with the beaver pelts*, *exported throughout the world*, *for they are only found on the northern rivers of the territory of the Rūs*, *the Bulghār and Kiev*” [5]. Where the Rus´ in eastern Europe seem to be central for the fur trade in the 9th to 10th centuries, the role of homeland Scandinavia is much more obscure. In Scandinavia, written sources suggest that fur derived from different sources. Some were local, some were paid in tribute by the Lapps of northern Scandinavia and sold on various Scandinavian markets [45], some came from Iceland [46] and some arrived via trade with the Rus´ [6]. Icelandic sagas [46] and the *History of the Archbishops of Hamburg-Bremen* by Adam of Bremen [47] testify to Scandinavian trade with eastern Europe and Novgorod. Adam of Bremen for instance mentions that Danes, under favourable conditions, could sail to Novgorod in one month [47]. Birka, in Sweden, was one of the most important Scandinavian market places and is in particular associated with the eastward trade in the 9th to 10th centuries [6]. It is not unlikely that the fur of wild animals that were no longer locally available were traded through Birka to the rest of Scandinavia. In the future, the analysis of stable isotopes may shed light on the relative prevalence of these routes and determine the provenance of individual objects [48].

### Fur, splendour and status

The importance of exclusive fur as part of elite splendour is well-known from both Arab and other western literary sources. Einhard’s description from the middle of the 9th century CE of the clothing of Charlemagne, for example, states: “*… he protected his shoulders and chest in winter by a close-fitting coat of otter or marten*”. [49]. In addition to the quote in the introduction by al-Mas´ūdī, this phenomenon is also described by Ibn Fadlān who, in 922 under a diplomatic travel to the Bulgars, witnessed a Viking ship burial and described how the noble Viking was buried in an elaborate outfit which included a cap covered with sable fur [5]. In the case of the beaver fur from the Hvilehøj and Bjerringhøj graves, there can be little doubt that these finds of clothing represent true traded luxury products aimed at displaying the magnificence of their owners. Probably most exported fur of wild species was so expensive, that it was only accessible to the elite. Beaver fur visually stands out from local furs by its sheen (S2 Fig in S1 File). It is moreover a heavy, very warm and water resistant fur. Local furs from marten and squirrel may have been more accessible and the local production of marten fur is also demonstrated through finds of marten bones with cut marks from flaying in bone assemblages from Fredshøj in Lejre and Ribe in Denmark [43]. Marten and squirrel fur is very light, but still warm, and based on its properties, not to mention the labour connected to catching the animals and preparing their much smaller skins, these furs must have still been exclusive and in high demand. Exclusive fur, such as the beaver fur found in Hvilehøj and Bjerringhøj, were easily recognisable compared to local wild and domestic resources and the wealth and power it signalled would have been understood by all the people in the Viking Age and placed the wearer into an international elite [43] with common markers of power.

How large a proportion of the population in the Viking Age who could wear clothing with luxury fur is still debatable. The Danish archaeological source material is heavily biased towards elite contexts where the burial styles have aided the preservation of organic materials, while graves that mirror more common people generally lack preserved fur. This bias forces us to pose the question of whether the lack of fur simply represents a taphonomic process or whether it was never there in the first place. Remains of textiles are found in graves belonging to lower socioeconomic individuals from the Scandinavian Viking Age [8], and thus one can argue that fur should also have been preserved if it was used in this context. Another possibility is that fur and skin materials in Danish Viking Age graves have not been systematically recorded and we currently lack knowledge of all fur objects recovered. In the well recorded sites of Birka and Hedeby, however, analysis shows that only few of the graves with high status markers as textile, silk and feathers have fur [50]. So based on this, the current lack of fur in more common graves seems genuine and shows an interesting facet of the use and trade of animal furs in Viking Age Denmark.

## Conclusion

The two proteomics methods applied in this paper were found to be in agreement and be superior to both the analysis of DNA and morphology through microscopy.

In this study we identify five examples of fur from one non-local species, beaver, in elite graves, providing evidence that fur trade and exotic furs played an important role in Viking Age Denmark. Importantly, this study also highlights that biomolecular identifications are limited by the publically available sequence databases, and efforts should be made to include more non-domesticated species to enable and increase the accuracy of future studies.

We show that fur from wild species in the chosen elite graves is connected to the clothing, whereas skin and leather from domesticated animals was mainly used for accessories including footwear and grave furnishing. The two materials thus seem to represent two different value chains. Based on the results presented here, fur was worn by the elite and by both sexes. It is also evident that fur and skin from several species were used for the same outfit, as seen in the Hvilehøj grave. This demonstrates extensive knowledge of the functionality and visual capacity of different skins, but also the wish to show off exclusive furs. Probably, wearing exclusive and imported fur placed the man from Bjerringhøj and the woman from Hvilehøj in the category of an international environment with shared social markers of wealth and power.

Thus, it is possible for the first time to see a more differentiated use of fur in the Viking Age, especially from wild animals which are connected with high status and luxury, which corresponds to values shown in the well-known Viking Age hunt for silver and other precious materials.

## Supporting information

S1 File Supporting information file containing supporting text, figures and tables. (DOCX)

S1 Dataset Containing tables of proteins and peptides identified by LC-MS/MS. (XLSX)
