# Supplementary material for: Palaeoproteomics identifies beaver fur in Danish high-status Viking Age burials - direct evidence of fur trade
Source: PLoS One. 2022 Jul 27;17(7):e0270040. doi: 10.1371/journal.pone.0270040 (PMC9328512; doi:10.1371/journal.pone.0270040)
Supplement: S1 File — (DOCX) [file pone.0270040.s001.docx]

**Supporting Information for**

Palaeoproteomics identifies beaver fur in Danish high-status Viking Age burials - direct evidence of fur trade

Luise Ørsted Brandt^1*^, Alberto J. Taurozzi^1^, Meaghan Mackie^1, 2^, Mikkel-Holger S. Sinding^3^, Filipe Garrett Vieira^1^, Anne Lisbeth Schmidt^4^, Charlotte Rimstad^4^, Matthew J. Collins^1^, and Ulla Mannering^4^

^1^ The GLOBE Institute, University of Copenhagen

^2^ The Novo Nordisk Foundation Center for Protein Research, University of Copenhagen

^3^ Department of Biology, University of Copenhagen

^4^ The National Museum of Denmark

Corresponding author: Luise Ørsted Brandt

Email: [luise.brandt@sund.ku.dk](mailto:xxxxx@xxxx.xxx)

**This PDF file includes:**

Supporting text

Figures S1 to S18

Tables S1 to S7

SI References

**Other supporting materials for this manuscript include the following:**

Datasets S1

**Table of content**

**Material**

Table S1. Contexts of the finds.

Fig S1. Figure of fur from all finds.

Fig S2. The appearance of marten, beaver, and squirrel fur.

**Methods**

Table S2. Overview of analytical strategy.

Text. DNA extraction and sequencing and analysis.

Table S3. Overview of DNA sequencing output.

Table S4. Table of reference mitogenomes used for mapping.

Text. Peptide mass fingerprinting of keratin.

Table S5. Observed keratin markers.

Fig S3-16. PMF spectra of samples not shown in the article.

Text. LC-MS of fur samples.

Table S6. Species identification based on LC-MS/MS.

Fig S17. Deamidation levels of samples and blanks.

Text. Microscopy of fur

Fig S18. Hairs observed in cross-sections and longitudinally mounted.

Table S7. Overveiw of identified species based on microscopic observations.

**Material**

**Table S1**. The selected finds and their contexts.

| **Site** | **Year of excavation** | **Dating of context** | **Context** | **Sex of deceased** | **Item** | **References** |
| --- | --- | --- | --- | --- | --- | --- |
| Hvilehøj | 1880 | 10th century | Grave. Waggon bed in burial mound | Female | Fragments of fur | [[1,2]](https://paperpile.com/c/Tol2oT/g0im4+2kisa/?locator=227%2C%20no.%20134,) |
| Bjerringhøj | 1868 | 970-971 | Grave. Wooden chamber in burial mound | Male | Fragments of fur from garment | [[1,3,4 no. 76,5]](https://paperpile.com/c/Tol2oT/g0im4+JpVMf+QDRKX+5LGFj/?locator=226%2C%20no.%20126,,102,&suffix=,,%20no.%2076,) |
| Skindbjerg | 1906 | 10th century | Grave. Wooden coffin in burial mound | Female | Fragments of fur | [[1,6]](https://paperpile.com/c/Tol2oT/g0im4+bpbp2/?locator=227%2C%20no.%20133,96%2C%20no.%2022) |
| Søllested | 1861 | 10th century | Grave. Chamber in burial mound | Unknown | Rolled up skin with fur | [[6,7]](https://paperpile.com/c/Tol2oT/ohLqM+bpbp2) |
| Ladby | 1934-37 | 10th century | Grave. Ship burial in burial mound | Male | Dog-harness, caulking/skin? | [[1,8,9]](https://paperpile.com/c/Tol2oT/g0im4+Iakrc+BHfVv/?locator=225%2C%20no.%2096,,) |
| Fyrkat | 1954-55 | 10th century | Grave | Female | Fur | [[10,11]](https://paperpile.com/c/Tol2oT/XRvYZ+eKfhz) |

**
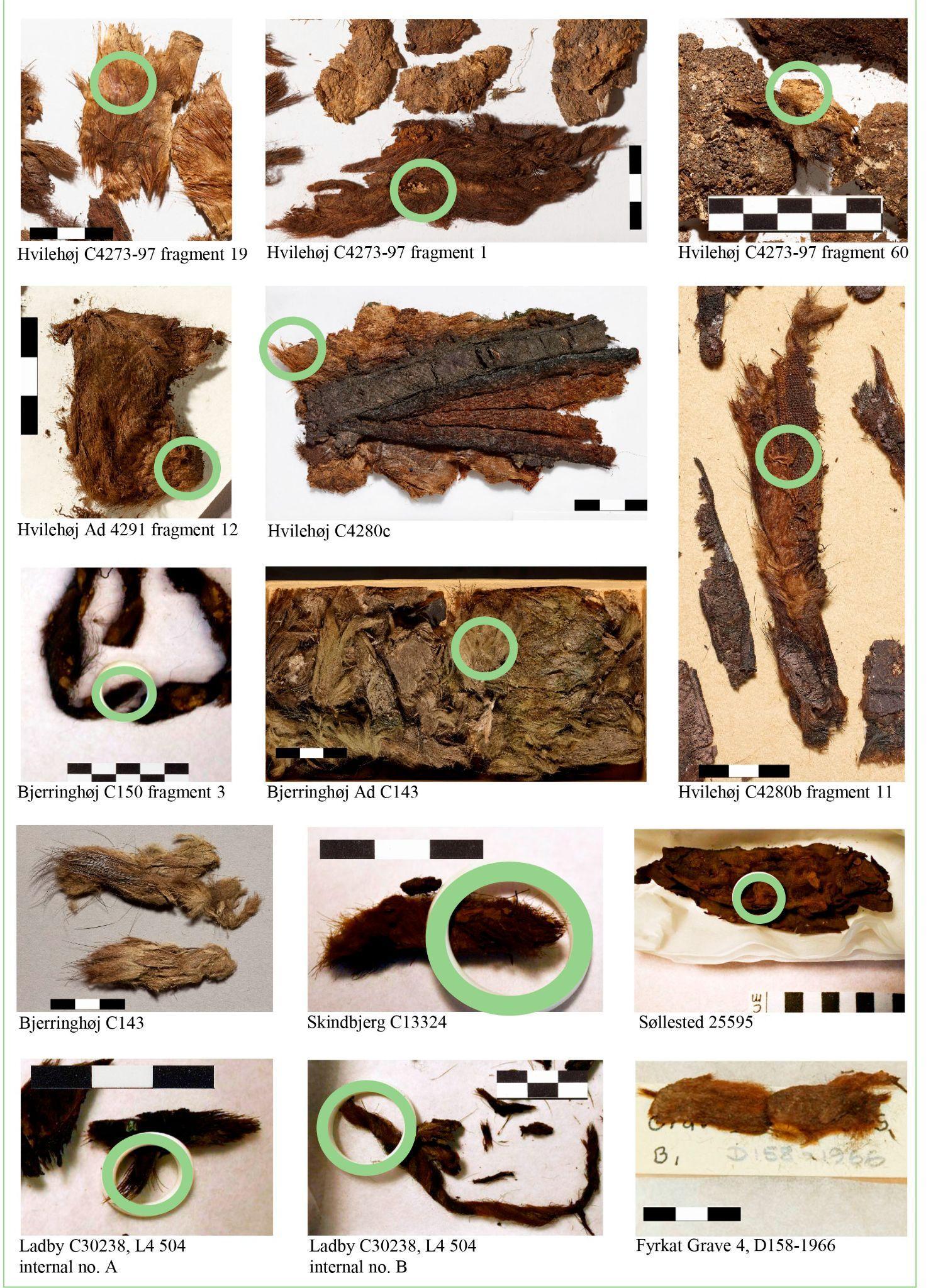
**

**Figure S1** The selected finds with sampling spots. Figure by Charlotte Rimstad.


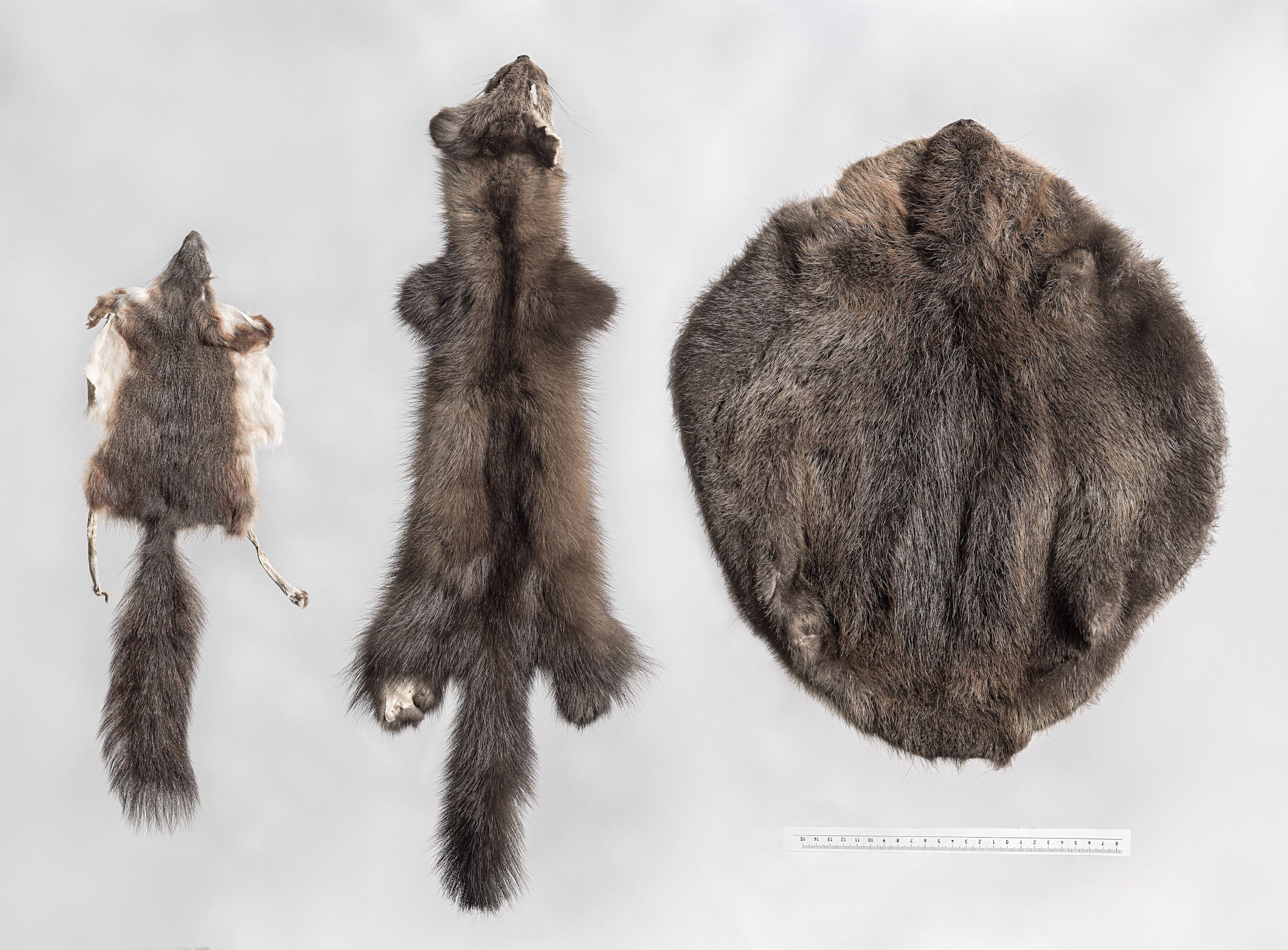


**Figure S2.** The physical appearance of from left: squirrel, marten, and beaver fur. Photos: Arnold Mikkelsen, the National Museum of Denmark.

**Methodology**

**Table S2.** Overview of analyses applied to each sample.

| **Site** | **Museum no.** | **aDNA** | **PMF** | **LC-MS** | **Microscopy** |
| --- | --- | --- | --- | --- | --- |
| Hvilehøj | C4273-97, fragment 19 | ✓ | ✓ | ✓ |  |
|  | C4273-97, fragment 1 | ✓ | ✓ | ✓ | ✓ |
|  | C4273-97, fragment 60 | ✓ | ✓ | ✓ | ✓ |
|  | AdC4291, fragment 12 | ✓ | ✓ |  | ✓ |
|  | C4280c | ✓ | ✓ |  | ✓ |
|  | C4280b, fragment 11 | ✓ | ✓ |  | ✓ |
| Bjerringhøj | C150, fragment 3 | ✓ | ✓ |  | ✓ |
|  | AdC143 | ✓ | ✓ | ✓ | ✓ |
|  | C143 | ✓ | ✓ |  | ✓ |
| Skindbjerg | C13324 | ✓ | ✓ |  | ✓ |
| Søllested* | 25595 | ✓ | ✓ |  | ✓ |
| Ladby | C30238, L4 504, internal no. A | ✓ | ✓ |  | ✓ |
| Ladby | C30238, L4 504, internal no. B | ✓ | ✓ |  | ✓ |
| Fyrkat | Grave 4, D158-1966, GuHCl protocol |  | ✓ | ✓ |  |
| Fyrkat | Grave 4, D158-1966, Urea protocol |  | ✓ | ✓ |  |

**Text. DNA extraction, sequencing and analysis**

DNA was extracted from 13 Viking Age fur samples and incorporated into Next Generation Sequencing (NGS) libraries in the clean laboratory facilities at the GLOBE Institute, following standard clean lab procedures [[12,13]](https://paperpile.com/c/Tol2oT/Jf7bt+N6Z9b).

Fur was digested in 2 ml DNA-lobind tubes (Eppendorf, Hamburg, Germany) using a buffer equal to Gilbert et al. [[14]](https://paperpile.com/c/Tol2oT/QTe1). In addition to the 13 fur samples, two extraction blanks of pure extraction buffer were incorporated in the workflow. The samples were pre-digested for one hour at 56 °C rotating 12 rounds per min, followed by a complete change of buffer and subsequently digestion-incubation for 12 hour under equal circumstances. The digest was centrifuged at 6.000 X g for 1 min to pellet solid remains. Approximately 1 ml of supernatant was removed and mixed 1:10, with a binding buffer as in [[15]](https://paperpile.com/c/Tol2oT/aAgDw), in a binding apparatus containing a Zymo-spin reservoir (Zymo Research, CA, USA) combined with a MinElute-spin column (Qiagen, Hilden, Germany) as in [[16]](https://paperpile.com/c/Tol2oT/V89Ca). The spin column was subsequently washed in 700 μl of PE buffer (Qiagen) and eluted by two steps of 20 μl EB buffer (Qiagen) in 1,5 ml DNA-lobind (Eppendorf) after 5 min incubation at 37 °C.

DNA was incorporated into NGS blunt-end library following the single-tube protocol of [[17]](https://paperpile.com/c/Tol2oT/XO8VC) with modifications following [[18]](https://paperpile.com/c/Tol2oT/CvZvx) and using Illumina-specific adapters [[19]](https://paperpile.com/c/Tol2oT/pOAXF). Indexing PCR was performed in a MJ Thermocycler (MJ Research, MA, USA), using PfuTurbo Cx Hotstart DNA Polymerase (Agilent Technologies) and Illumina's (Illumina, CA, USA) Multiplexing PCR primer (1,5 μM of inPE1.0 5′ AATGATACGGCGACCACCGAGATCTACACTCTTTCCCTACACGACGCTCTTCCGATCT with a custom-designed index primer with a six nucleotide index 5′-CAAGCAGAAGACGGCATACGAGATNNNNNNGTGACTGGAGTTC). Library amplifications were quantified using an Agilent 2100 Bioanalyzer (Agilent Technologies, CA, USA) using a HS chip and reagents. The samples were finally pooled in equimolar amounts and sequenced at the Danish National High-Throughput DNA Sequencing Centre on the Illumina HiSeq 2500 platform (Illumina), using one lane 100bp single read chemistry. In addition to the samples and extraction blanks, further two library blanks were incorporated in the workflow, using molecular grade H^2^O instead of library.

Sequencing reads were processed and mapped against a diverse dataset of 68 reference mitochondrial sequences using the PALEOMIX pipeline [[20]](https://paperpile.com/c/Tol2oT/TYYWX). The reference sequences included species mentioned in the written sources [[21,22]](https://paperpile.com/c/Tol2oT/EuICH+xdKkS) and other species in these genera present in the local area (Table S4). Following a mitochondrial consensus sequence for each sample against each reference was produced using ANGSD [[23]](https://paperpile.com/c/Tol2oT/VgF7l) as in [[24]](https://paperpile.com/c/Tol2oT/gAW4V).

**Table S3.** Overview of sequencing output and mapping details for each sample. The “best match” is the reference sequence to which the highest number of unique reads “hits_unique (best match)” was mapped. The amount of reads mapping to any reference is extremely low, allowing no interpretation of genetic affiliation.

| **#Sample_ID** | **Retained Reads** | **Retained Nucleotides (bps)** | **Average Read Length (bps)** | **Best Species Match** | | | | | | | |
| --- | --- | --- | --- | --- | --- | --- | --- | --- | --- | --- | --- |
|  |  |  |  | **Species** | **Raw Hits** | **Fraction Raw Hits** | **Hits Clonality (%)** | **Unique Hits** | **Fraction Unique Hits** | **Hits Coverage** | **Average Hits Length (bps)** |
| Bjerringhøj C143 | 1,395,422 | 82,138,972 | 58.86 | Homo sapiens | 61 | 4,37E-05 | 92 % | 5 | 3,58E-06 | 0,0201 | 66,60 |
| Bjerringhøj C150, fragment 3 | 1,014,051 | 68,105,321 | 67.16 | Homo sapiens | 148 | 0,000145949 | 70 % | 44 | 4,34E-05 | 0,1759 | 66,27 |
| Bjerringhøj, AdC143 | 1,621,680 | 101,346,378 | 62.49 | Homo sapiens | 134 | 8,26E-05 | 82 % | 24 | 1,48E-05 | 0,0899 | 62,04 |
| Hvilehøj C4273-97, fragment 19 | 1,044,197 | 47,511,503 | 45.50 | Gulo gulo | 14 | 1,34E-05 | 21 % | 11 | 1,05E-05 | 0,0295 | 44,45 |
| Hvilehøj C4273-97, fragment 1 | 1,727,310 | 74,372,386 | 43.06 | Gulo gulo | 27 | 1,56E-05 | 33 % | 18 | 1,04E-05 | 0,0486 | 44,72 |
| Hvilehøj C4273-97, fragment 60 | 7,351,856 | 351,433,120 | 47.80 | Gulo gulo | 9 | 1,22E-06 | 33 % | 6 | 8,16E-07 | 0,017 | 48,00 |
| Hvilehøj AdC4291, fragment 12 | 1,475,843 | 63,766,103 | 43.21 | Homo sapiens | 1 | 6,78E-07 | 0 % | 1 | 6,78E-07 | 0,0021 | 34,00 |
| Hvilehøj C4280c | 4,662,849 | 204,331,073 | 43.82 | Homo sapiens | 18 | 3,86E-06 | 61 % | 7 | 1,50E-06 | 0,0258 | 61,00 |
| Hvilehøj C4280b, fragment 11 | 3,009,142 | 185,791,008 | 61.74 | Homo sapiens | 21 | 6,98E-06 | 33 % | 14 | 4,65E-06 | 0,0567 | 67,14 |
| Ladby C30238, internal no. A | 1,425,466 | 90,846,101 | 63.73 | Gulo gulo | 28 | 1,96E-05 | 79 % | 6 | 4,21E-06 | 0,0135 | 37,33 |
| Ladby C30238, internal no. B | 1,740,459 | 104,333,023 | 59.95 | Gallus gallus | 123 | 7,07E-05 | 98 % | 2 | 1,15E-06 | 0,0070 | 59,00 |
| Skindbjerg C13324 | 1,934,555 | 99,065,407 | 51.21 | Martes zibellina | 1 | 5,17E-07 | 0 % | 1 | 5,17E-07 | 0,0049 | 81,00 |
| Søllested 25595, fur | 1,383,362 | 85,852,444 | 62.06 | Gulo gulo | 1 | 7,23E-07 | 0 % | 1 | 7,23E-07 | 0,0018 | 30,00 |

**Table S4.** Table of reference mitogenomes used for mapping our samples and NCBI acc. no.

| **Species** | **Reference sequence** |
| --- | --- |
| *Alces alces* | NC020677 |
| *Anas platyrhynchos* | NC009684 |
| *Anser anser* | NC011196 |
| *Bison bison* | EU177871 |
| *Bison bonasus* | NC014044 |
| *Bos indicus* | JN817298 |
| *Bos primigenius* | NC013996 |
| *Bos taurus* | KC153977 |
| *Callorhinus ursinus* | NC008415 |
| *Canis lupus* | KF661049 |
| *Capra hircus* | NC005044 |
| *Capreolus capreolus* | NC020684 |
| *Castor fiber* | NC028625 |
| *Cervus elaphus* | KP172593 |
| *Chroicocephalus ridibundus* | NC025649 |
| *Cynomys leucurus* | KP326309 |
| *Cynomys ludovicianus* | KP326310 |
| *Cystophora cristata* | NC008427 |
| *Dama dama* | NC020700 |
| *Enhydra lutris* | NC009692 |
| *Equus caballus* | NC001640 |
| *Erignathus barbatus* | NC008426 |
| *Eumetopias jubatus* | AB300608 |
| *Felis catus* | NC001700 |
| *Gallus gallus* | NC001323 |
| *Gulo gulo* | NC009685 |
| *Halichoerus grypus* | X72004 |
| *Homo sapiens* | NC012920 |
| *Ictidomys tridecemlineatus* | KP698974 |
| *Lepus americanus* | KJ397613 |
| *Lepus arcticus* | KJ397607 |
| *Lepus californicus* | KJ397614 |
| *Lepus capensis* | KJ397612 |
| *Lepus corsicanus* | KJ397606 |
| *Lepus granatensis* | KJ397610 |
| *Lepus granatensis* | KJ397611 |
| *Lepus othus* | KJ397608 |
| *Lepus townsendii* | KJ397609 |
| *Lepus europaeus* | NC004028 |
| *Lepus timidus* | KJ397605 |
| *Lutra lutra* | NC011358 |
| *Lynx lynx* | NC027083 |
| *Marmota himalayana* | NC018367 |
| *Martes martes* | NC021749 |
| *Martes zibellina* | NC011579 |
| *Meles meles* | NC011125 |
| *Mustela erminea* | NC025516 |
| *Mustela nivalis* | NC020639 |
| *Mustela putorius* | NC020638 |
| *Odobenus rosmarus* | AJ428576 |
| *Oryctolagus cuniculus* | NC001913 |
| *Ovibos moschatus* | FJ207536 |
| *Ovis aries* | NC001941 |
| *Pagophilus groenlandicus* | AM181030 |
| *Phoca fasciata* | NC008428 |
| *Phoca largha* | FJ895151 |
| *Phoca vitulina* | X63726 |
| *Pusa hispida* | NC008433 |
| *Pusa sibirica* | NC008432 |
| *Rangifer tarandus* | KM506758 |
| *Sciurus vulgaris* | NC002369 |
| *Sus scrofa* | NC000845 |
| *Tamias sibiricus* | NC025277 |
| *Ursus americanus* | AF303109 |
| *Ursus arctos* | AF303110 |
| *Ursus maritimus* | AF303111 |
| *Vulpes lagopus* | NC026529 |
| *Vulpes vulpes* | NC008434 |

**Text. Peptide mass fingerprinting of keratin.**

**Sampling, protein extraction, digestion and peptide purification**

The protocol used was based on the method published by Solazzo et al. [[25]](https://paperpile.com/c/Tol2oT/33KSS/?noauthor=1). Initially fur samples were chopped into short hair strands and washed three times using molecular biology grade water in an eppendorf by adding 1mL water, vortexing for 1 minute and removing the supernatant. Subsequently the samples were suspended in 200 µL of lysis buffer (8M Urea, 100 mM Ammonium Bicarbonate (Sigma Aldrich) and 50 mM TCEP (Sigma Aldrich) adjusted to pH in the optimum of trypsin 8-9 with ammonium hydroxide (Sigma Aldrich)) following [[25]](https://paperpile.com/c/Tol2oT/33KSS). They were incubated for 3 hours shaking at 65 °C. Protein quantification was performed using the Bradford assay.

Samples were alkylated for 45 min at room temperature in the dark using iodoacetamide (IAM) (Sigma Aldrich) at a final concentration of 40 mM. 20ug of protein from each sample was then diluted fivefold with 50 mM Ammonium Bicarbonate (pH 8.4), and digestion was carried at 37 °C overnight with 1 µg of trypsin (Promega) per sample. Following digestion, samples were evaporated to dryness using a centrifugal evaporator (Eppendorf, Hamburg, Germany) and re-solubilised in 50 µL of a 0.1% TFA (Sigma Aldrich) aqueous solution. Samples were acidified with 50% Trifluoroacetic acid (TFA, Sigma Aldrich) to below pH 2. Peptide purification was performed using 100 µL Pierce™ C18 Tips containing reversed-phase C18 resin. The tips were washed first twice with 50%/50% acetonitrile/0.1% TFA and then twice with 0.1% TFA. After loading the samples, the tips were washed twice with 0.1% TFA and the samples eluted with 50 µL of 50%/50% acetonitrile (ACN)/0.1% TFA.

Note samples from Ladby C30238, L4 504 A and B and Skindbjerg C13324 were previously extracted using Ambic without reduction and alkylation [[26]](https://paperpile.com/c/Tol2oT/fck3M) as part of a previous study (unpublished). The leftover pellets from the original extraction were processed as described above.

**GuHCl extraction**

Fur samples were chopped into small pieces using scissors and washed three times using molecular biology grade water in an eppendorf by adding 1mL water, vortexing for 1 minute and removing the supernatant. 50-100ul (volume sufficient to submerge the sample) of extraction buffer (4M GuHCl, 25mM TRIS, 10mM TCEP, 20mM CAA) was added and the samples were incubated at 95℃ for 30 minutes with shaking at 1500rpm. Protein quantification was performed using a reducing agent compatible BCA kit according to manufacturer's instructions. A 5-point standard curve was included and results were read using a plate reader. A volume of supernatant equivalent to 20ug of protein was removed into a new eppendorf tube and diluted to a final GuHCl concentration of 0.6M using 50mM Ammonium Bicarbonate (pH . 8.4). 0.4ug of trypsin was added (enzyme:protein ratio = 1:50) and digestion was performed for 18h at 37℃ with shaking at 750rpm. Following digestion, peptide purification was performed using 100 µL Pierce™ C18 Tips containing reversed-phase C18 resin. The tips were washed first twice with 50%/50% acetonitrile/0.1% TFA and then twice with 0.1% TFA. After loading the samples, the tips were washed twice with 0.1% TFA and the samples eluted with 50 µL of 50%/50% acetonitrile (ACN)/0.1% TFA.

**Peptide mass fingerprinting of keratin**

Peptide eluates were co-crystallised onto a Bruker steel plate with α-cyano-4-hydroxycinnamic acid (Sigma Aldrich) matrix solution (50% ACN /0.1% TFA (vol/vol) at a ratio of 1:1 (1 μL : 1 μL). The plate was sent to BioArCh, University of York, for MALDI-ToF analysis. The samples were analysed on a Bruker ultrafleX III MALDI-ToF instrument in reflector mode, the laser intensity was set between 40–50% and the mass range was set at 800–4000 Da. Peptide masses below 650 Da were suppressed. Each sample was externally calibrated against an adjacent spot containing a mixture of six peptides (des-Arg1 Bradykinn m/z=904.681, Angiotensin I m/z= 1295.685, Glu1-Fibrino-peptide B m/z= 1750.677, ACTH (1–17 clip) m/z=2093.086, ACTH (18–39 clip) m/z= 2465.198 and ACTH (7–38 clip) m/z= 3657.929). Spectral analysis was performed by the authors using the open-source cross-platform software mMass (www.mmass.org) [[27]](https://paperpile.com/c/Tol2oT/2ORgY). The three spectra generated for each sample replicate were averaged (available at PRIDE identifier PXD030529), and the average spectrum was inspected manually for the presence of peptide markers [[28–30]](https://paperpile.com/c/Tol2oT/QhVNq+UbrNz+ktnJy). Spectra of poor quality (i.e. low signal-to-noise (S/N) ratio and no distinct markers) were designated ‘No ID’.

Diagnostic peptide markers allow for identification of keratinous materials to genus level [[28]](https://paperpile.com/c/Tol2oT/QhVNq) and the reference material at the present includes several domesticated species, as well as species within the families of Cervidae, Camelidae, Equidae, Leporidae, Candae, and Phocidae [[31]](https://paperpile.com/c/Tol2oT/CG7t). The database has recently been expanded with a wide range of fur-bearing animals of North America familie [[29]](https://paperpile.com/c/Tol2oT/UbrNz). Peptide markers were compared with published markers for mammals [[28,29]](https://paperpile.com/c/Tol2oT/UbrNz+QhVNq) following the order described by Solazzo in Figure 2 [[29]](https://paperpile.com/c/Tol2oT/UbrNz). Taxonomic identifications were assigned at the most conservative level of identification (genus or family level) based on the presence of unambiguous markers (marked ‘?’ for low intensity, or low S/N threshold).

Note samples from Ladby C30238, L4 504, internal numbers A and B and Skindbjerg C13324 were treated as above but sent for MALDI-TOF analysis at University of Cambridge, Department of Archaeology instead of York. Here, samples were analysed using a Bruker ultrafleXtrem MALDI-TOF mass spectrometer (Department of Chemistry, University of Cambridge, UK) with a smartbeam-II laser. The MALDI was operated in reflector mode with a m/z range of 798–3985 Da.

**Table S5.** Observed PMF markers in the samples. *Possible human contamination.

| **Lokalitet** | **M. no.** | I-L_2A | I-L_2B | I-2B(a) | I-2B(b) | I-2B(c) | I-2B(d) | II-1A_L | II-1B | II-L_2B | II-2B(a) | II-2B(b) | **PMF ID** |
| --- | --- | --- | --- | --- | --- | --- | --- | --- | --- | --- | --- | --- | --- |
| Hvilehøj | C4273-97, fragment 19 | 1669,87 | 1109,53 | - | 1848,99 | 2593,35* | 1504,77 | 1041,49 | 2007,99; 2050,99; 2179,09 | 1011,45 | 2088,02 | 1263,69 | *Castor* |
| Hvilehøj | C4273-97, fragment 1 | 1625,85? | 1109,53 | - | 1834,98 | 2577,30 | 1504,77 | - | - | - | - | 1263,69 | Bovidae/cervidae |
| Hvilehøj | C4273-97, fragment 60 | 1625,85; 1669,87 | 1109,53 | - | 1848,99 | 2595,31; 2639,28? | 1504,77; 1518,79 | 1041,49 | 2050,99; 2163,09; 2179,09 | 1011,45 | 2104,02 | 1263,69 | Closest match to *Castor* |
| Hvilehøj | AdC4291, fragment 12 | 1625,85; 1669,87 | 1109,53 | - | 1848,99 | - | 1504,77 | 1041,49 | 2050,99; 2163,09; 2179,09 | 1011,45? | 2088,02? | 1263,69 | *Castor* |
| Hvilehøj | C4280c | 1625,85; 1669,87? | 1109,53 | - | 1848,99 | 2595,31 | 1504,77?; 1518,79? | - | 2163,09; 2179,09? | - | 2104,02? | 1263,69 | Closest match to *Castor* |
| Hvilehøj | C4280b, fragment 11 | 1669,87 | 1109,53 | - | 1848,99 | - | 1504,77 | 1041,49 | 2050,99; 2179,09? | - | 2088,02? | 1263,69 | *Castor* |
| Bjerringhøj | C150, fragment 3 | 1625,85 | 1109,53 | - | 1834,98 | - | 1504,77 | - | 2191,12; 2063.03? | 1011,45 | 2113,05? | 1263,69 | Bovidae/cervidae |
| Bjerringhøj | AdC143 | 1669,87 | 1109,53 | - | 1848,99 | - | 1504,77 | 1041,49 | 2163,09; 2179,09 | 1011,45 | 2088,02 | 1263,69 | *Castor* |
| Bjerringhøj | C143 | 1669,87 | 1109,53 | - | 1848,99 | - | 1504,77 | 1041,49 | 2163,09?; 2179,09 | 1011,45? | 2088,02 | 1263,69 | *Castor* |
| Skindbjerg | C13324 | - | - | - | - | - | - | - | - | - | - | - | No ID |
| Søllested‡ | 25595 | - | - | - | - | - | - | - | - | - | - | 1263,69? | No ID |
| Ladby | C30238, L4 504, A | 1625,85, 1669,87 | 1109,53 | - | 1834,98 | - | - | - | - | 1011,45 | - | 1263,69 | Bovidae/cervidae? |
| Ladby | C30238, L4 504, B | 1625,85, 1669,87 | - | - | 1848,99 | - | - | - | - | 1011,45 | - | 1263,69 | No ID |
| Fyrkat | Grave 4, D158-1966, Urea protocol | 1625,85 | 1109,53 | - | 1848,99 | - | - | - | 2035,00/2035,98?, 2164,08 | 1011,45? | 2088,02, 2104,02? | 1263,69 | Mustelidae/Ursidae |
| Fyrkat | Grave 4, D158-1966, GuHCl protocol | - | - | - | 1848,99 | - | - | - | 2035,00/2035,98?, 2164,08 | - | 2088,02, 2104,02? | 1263,69 | Mustelidae/Ursidae |


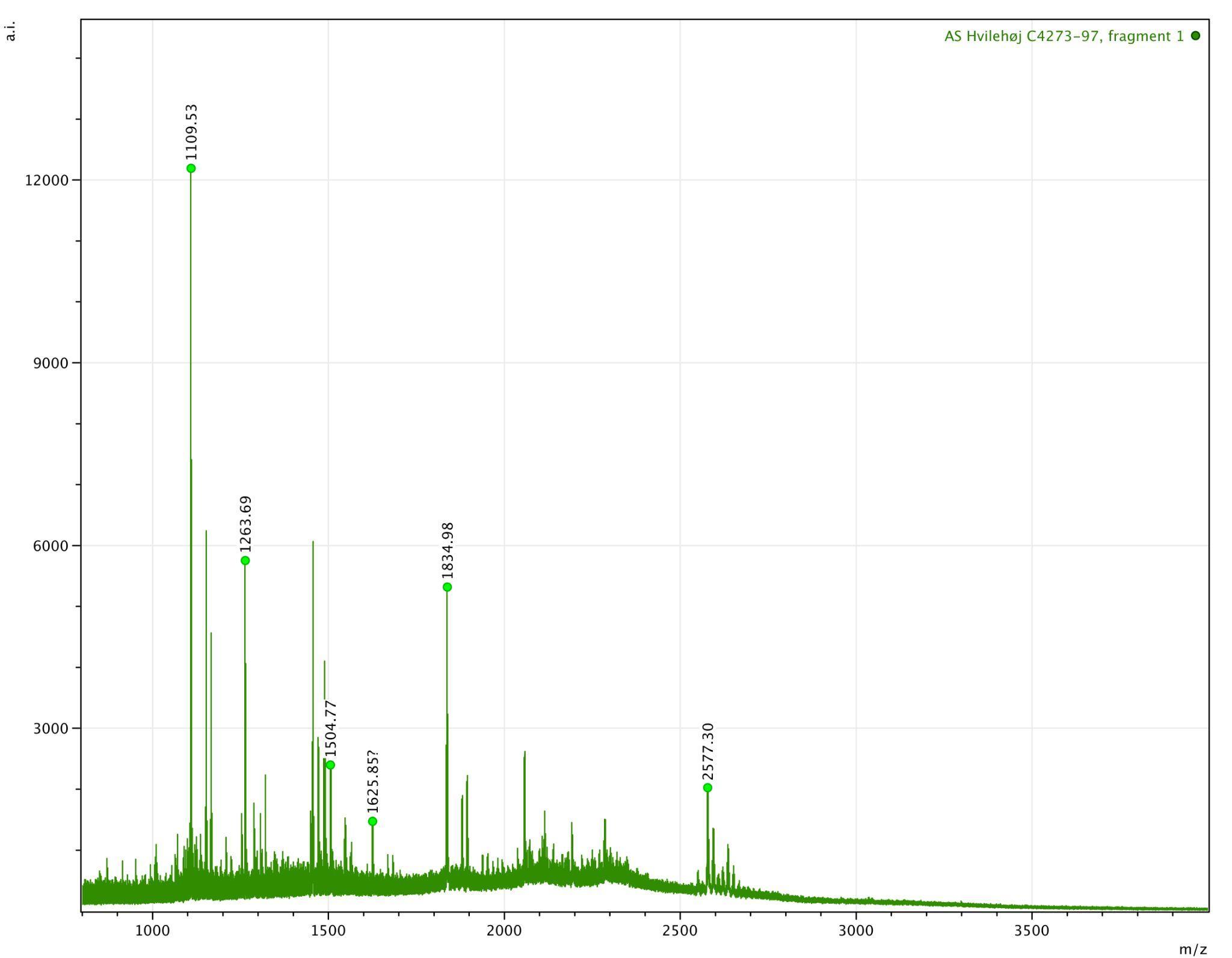


**Figure S3.** Averaged spectrum (AS) with markers for Hvilehøj C4273-97, fragment 1.


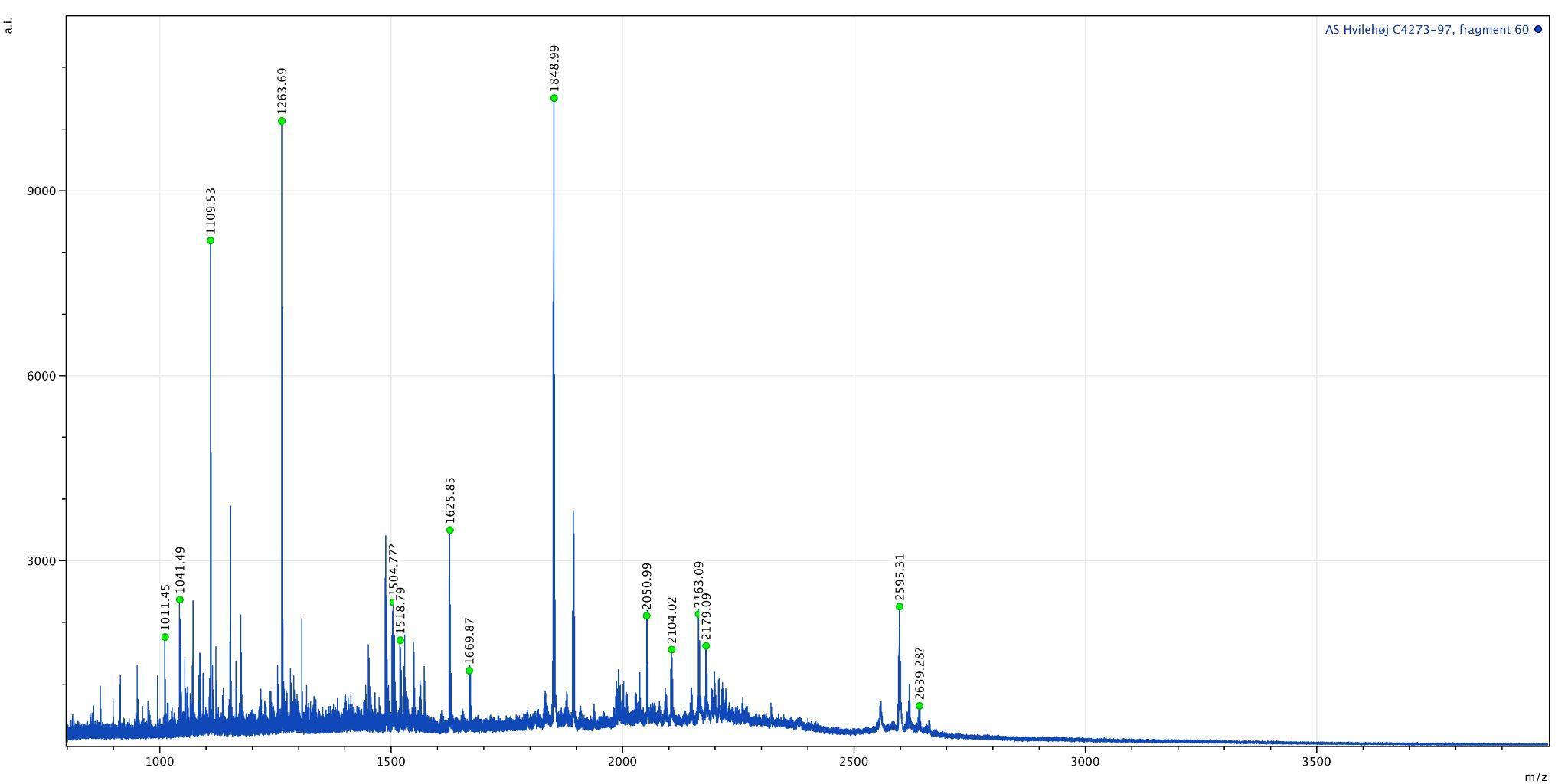


**Figure S4.** Averaged spectrum (AS) with markers for Hvilehøj C4273-97, fragment 60.


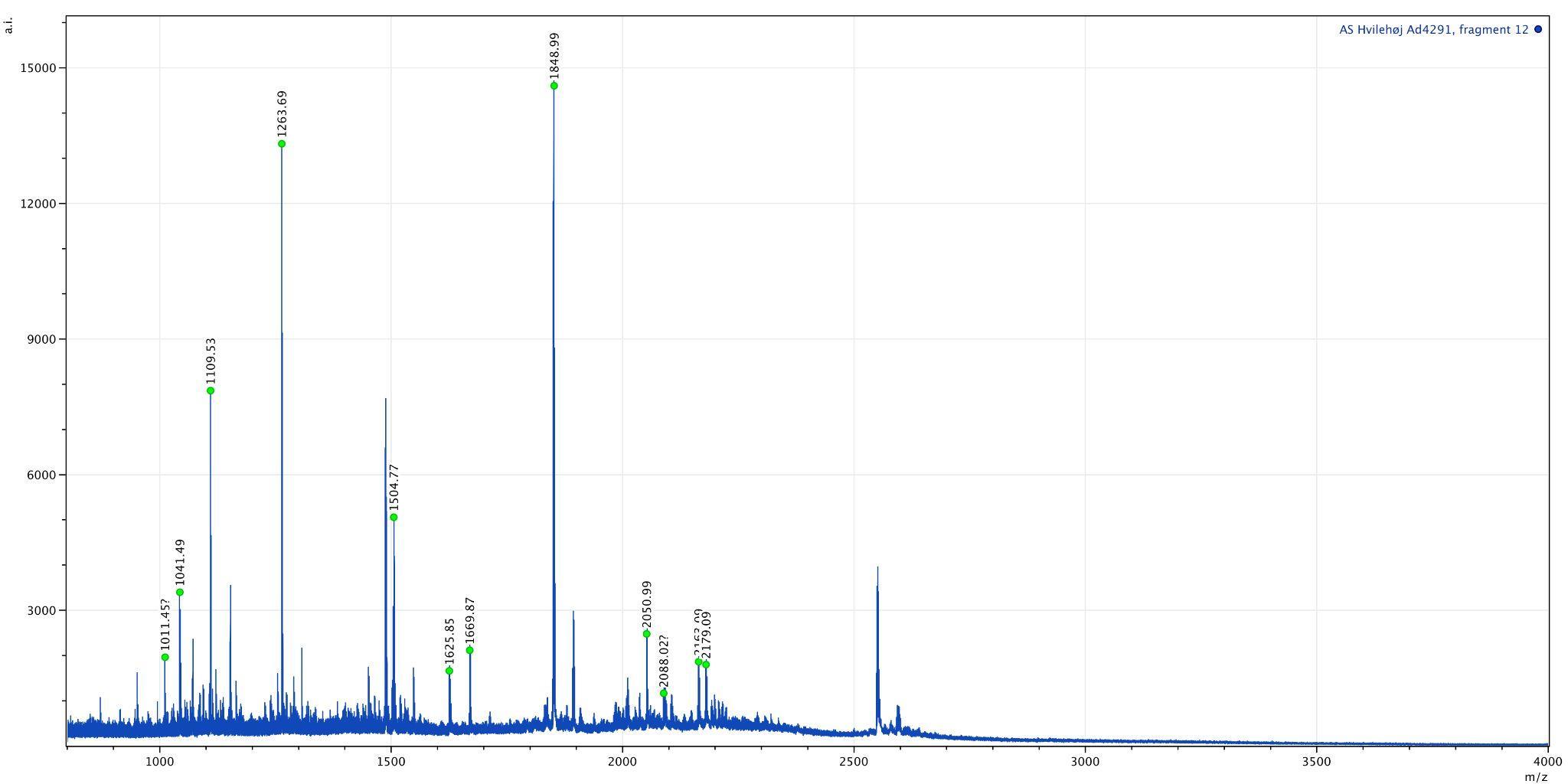


**Figure S5.** Averaged spectrum (AS) with markers for Hvilehøj AdC4291, fragment 12.


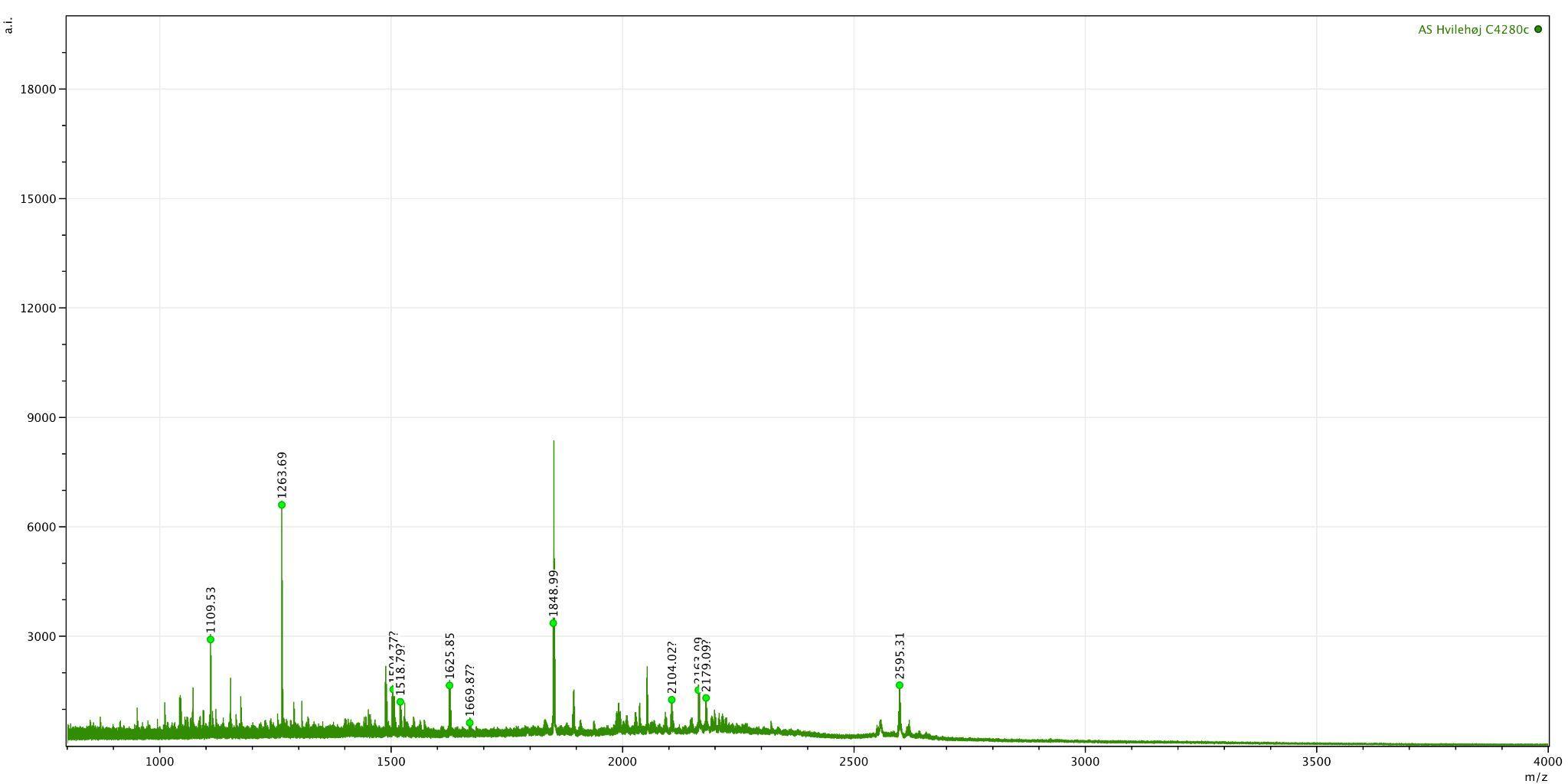


**Figure S6.** Averaged spectrum (AS) with markers for Hvilehøj C4280c.


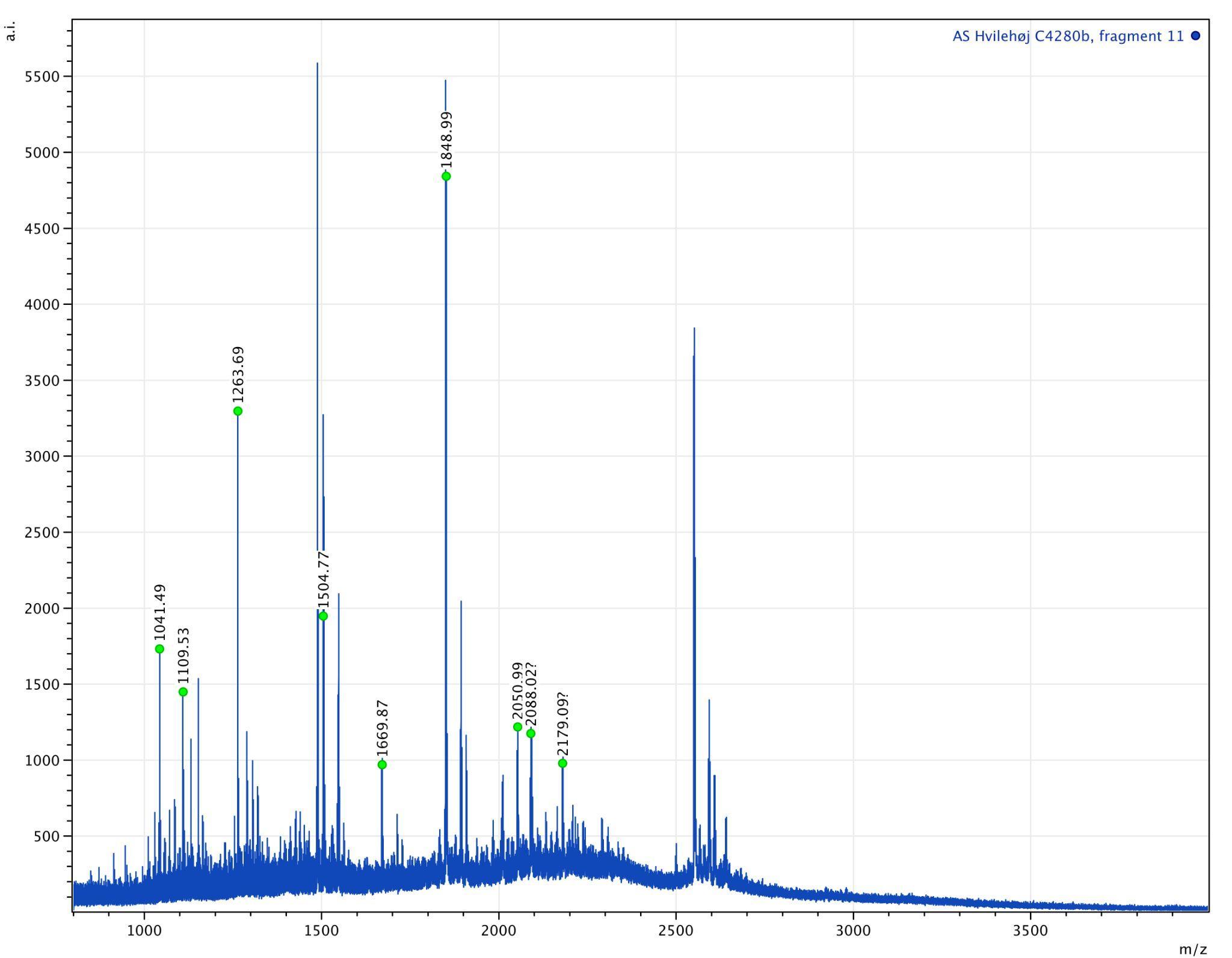


**Figure S7.** Averaged spectrum (AS) with markers for HvilehøjC4280b, fragment 11.

**
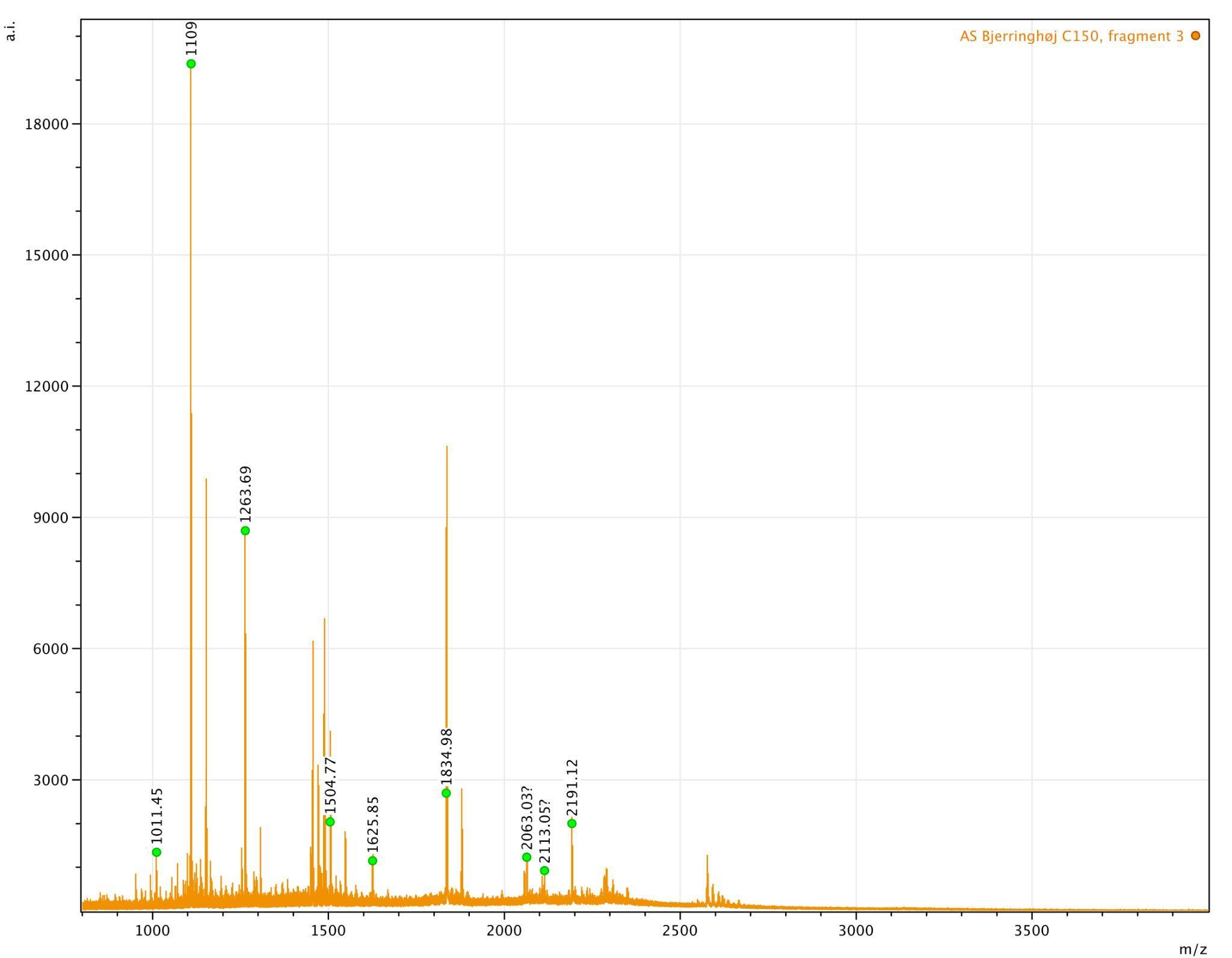
**

**Figure S8.** Averaged spectrum (AS) with markers for Bjerringhøj, C150, fragment 3.


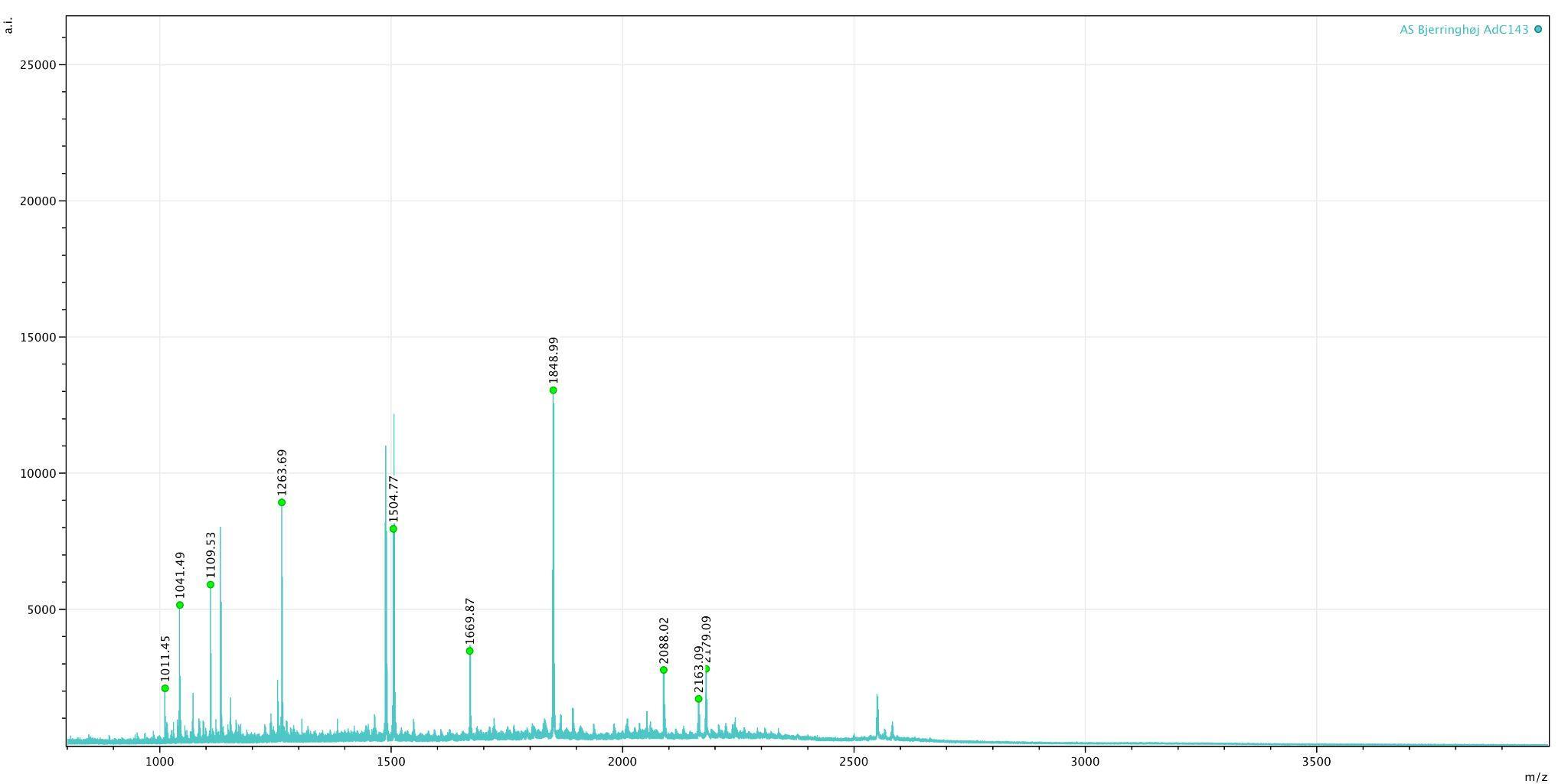


**Figure S9.** Averaged spectrum (AS) with markers for Bjerringhøj, AdC143.


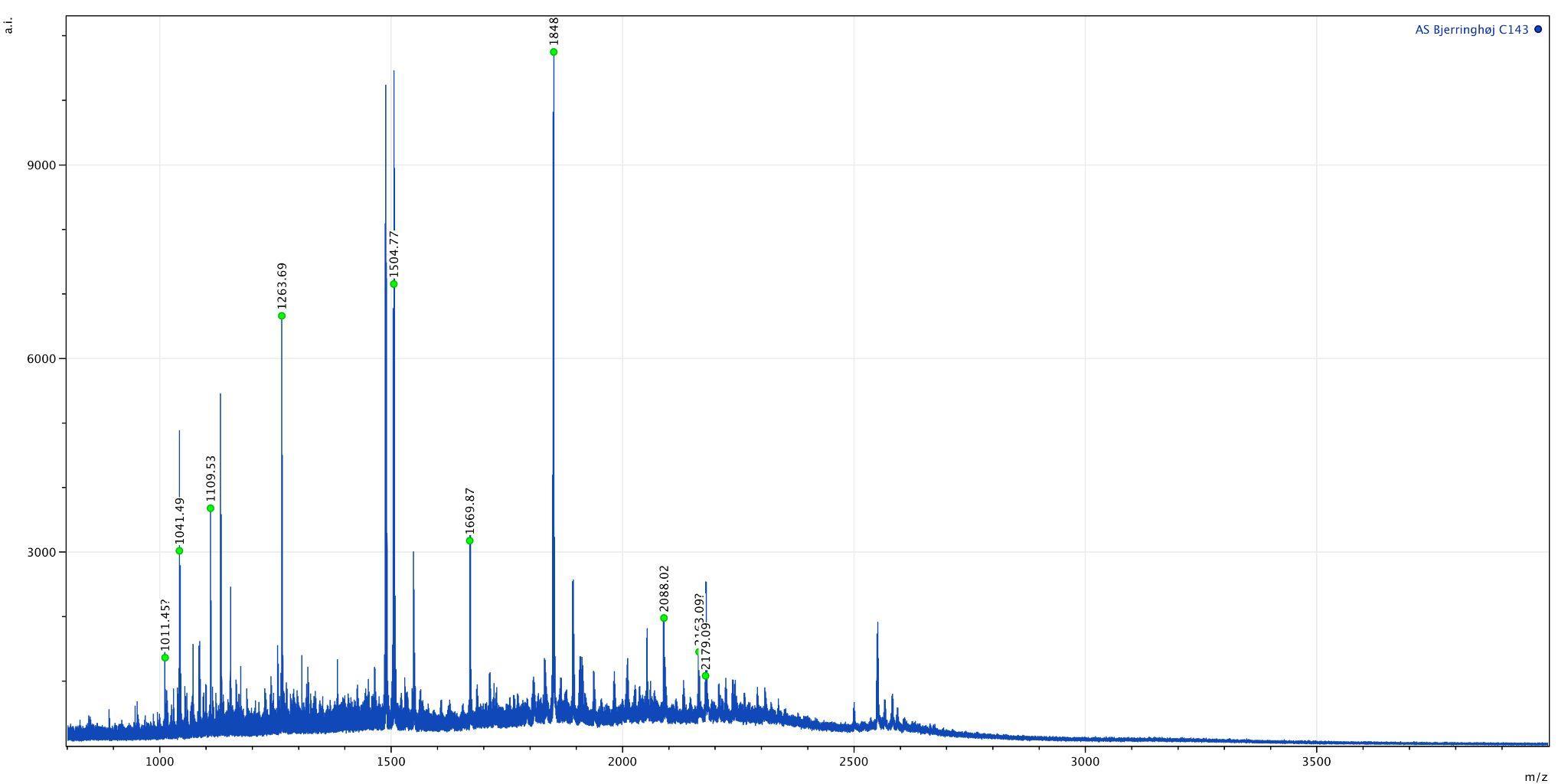


**Figure S10.** Averaged spectrum (AS) with markers for Bjerringhøj C143.


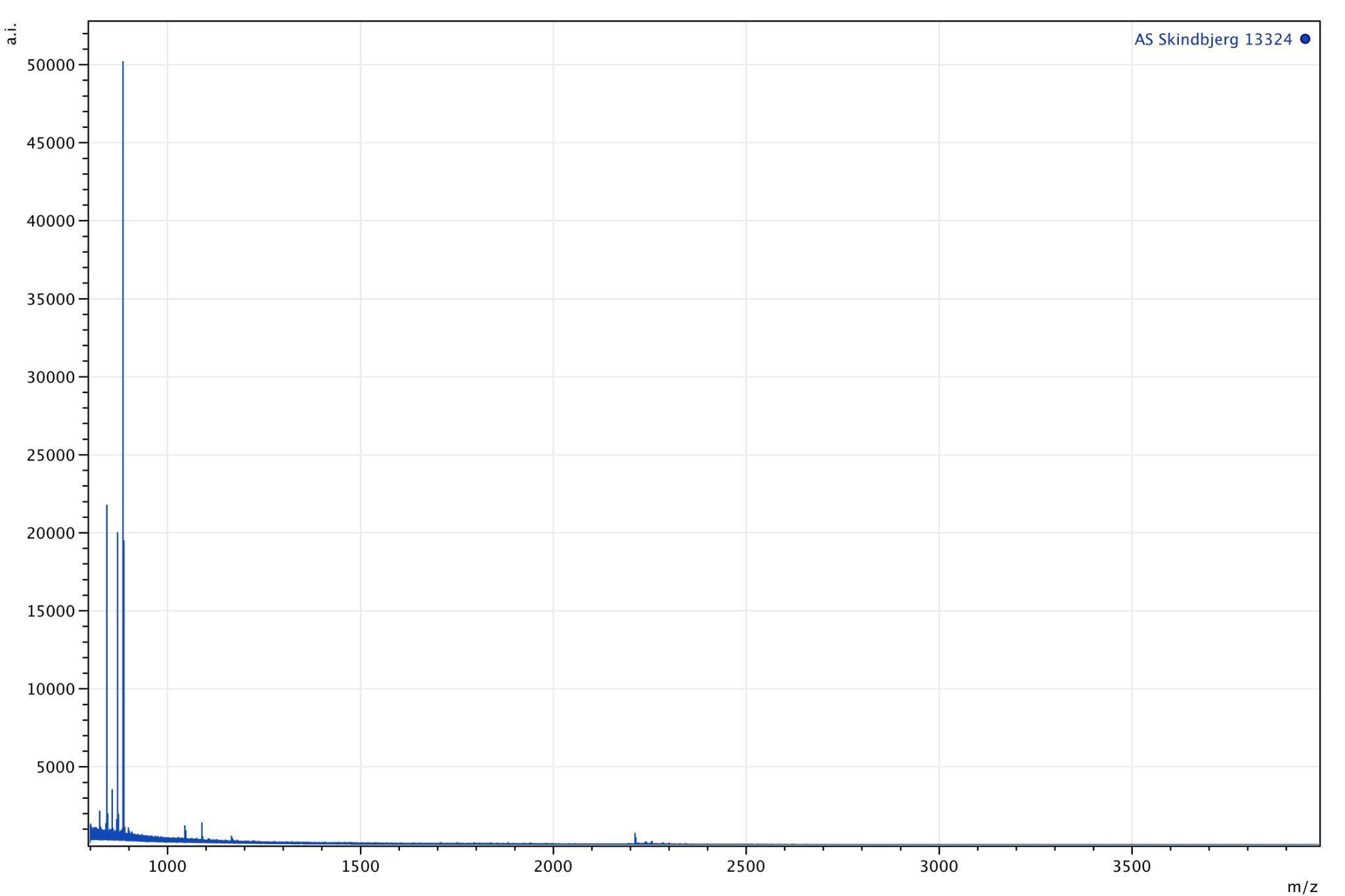


**Figure S11.** Averaged spectrum (AS) with markers for Skindbjerg 13324.


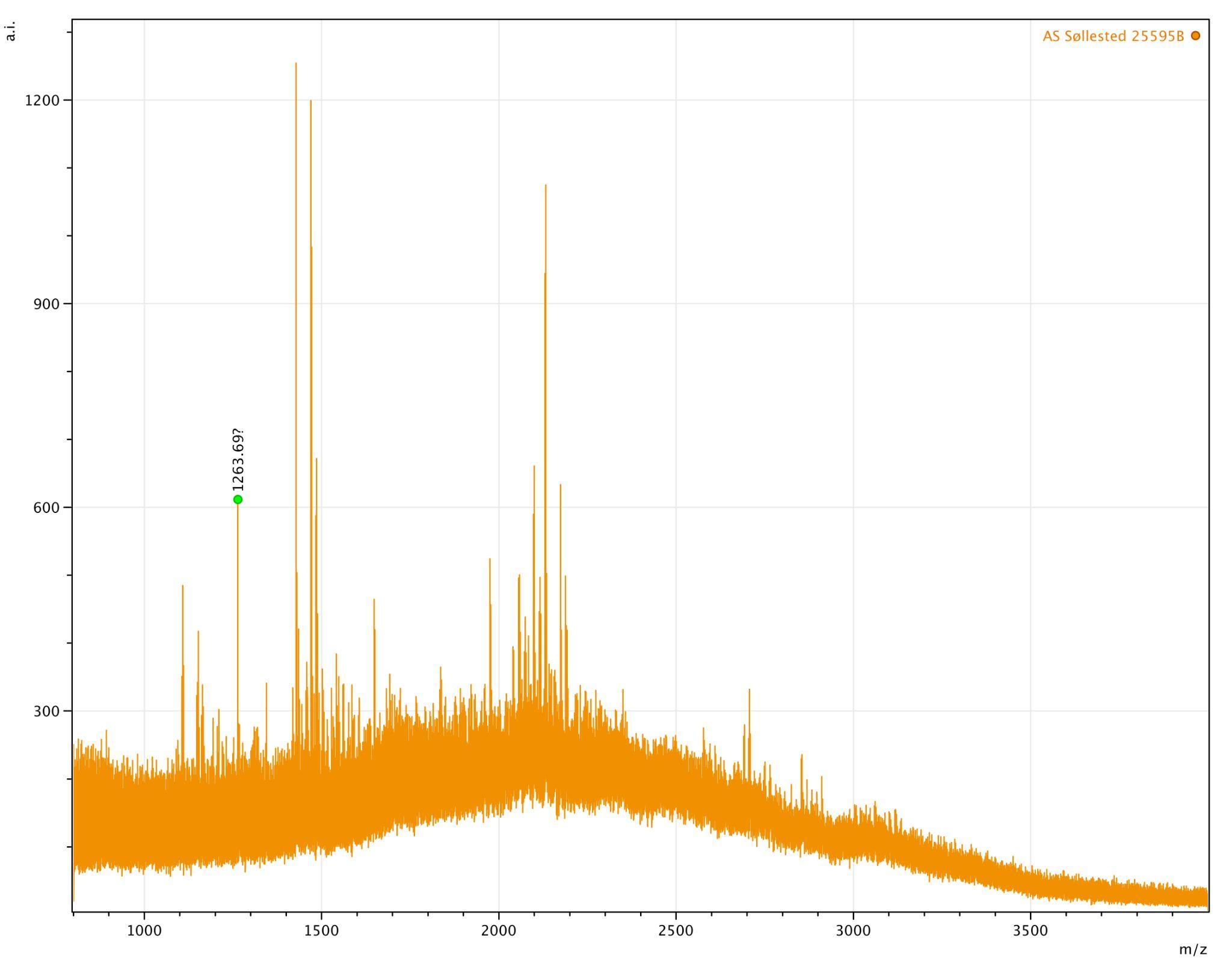


**Figure S12.** Averaged spectrum (AS) with markers for the fur sampled from Søllested 25595.

.
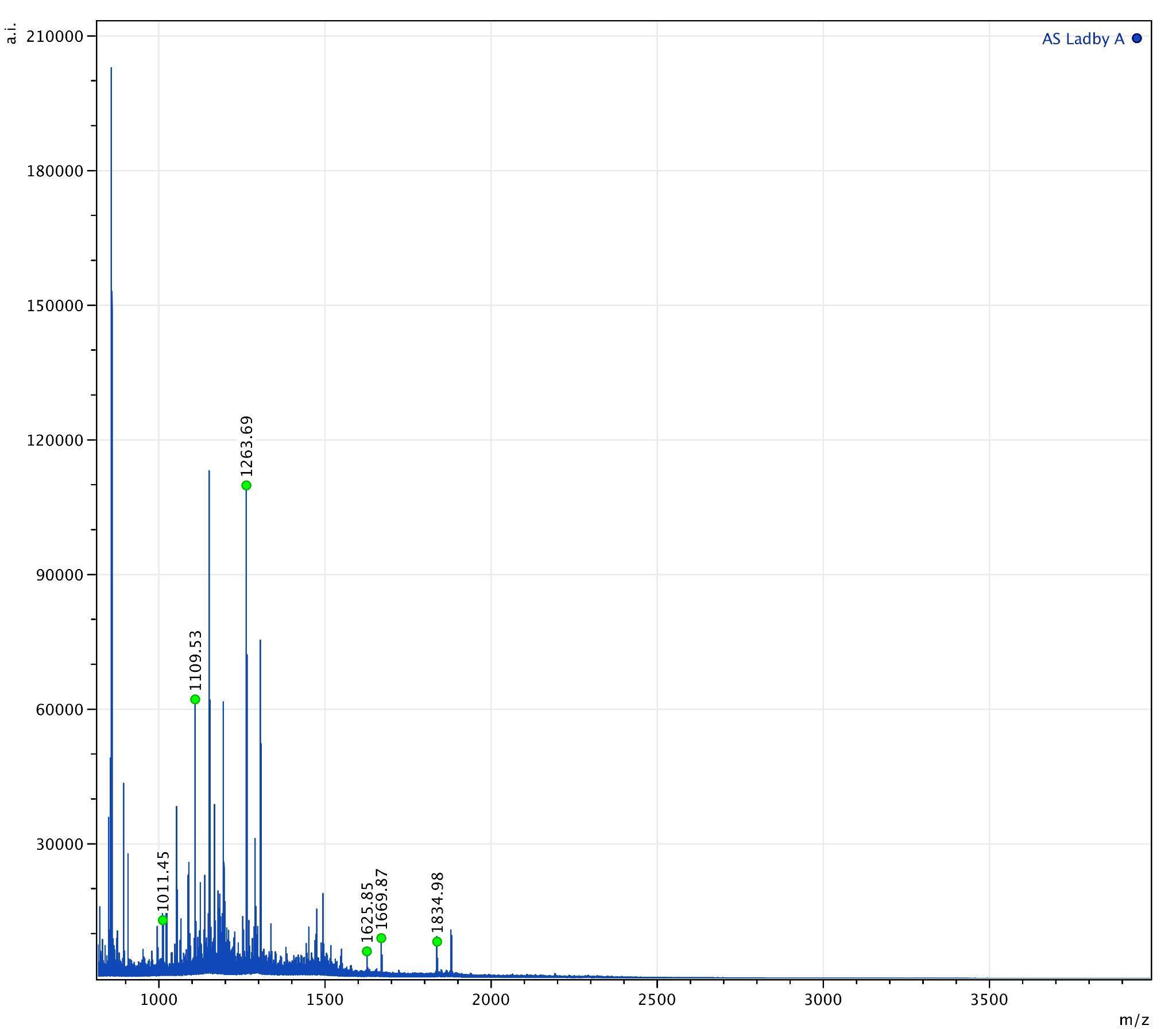


**Figure S13.** Averaged spectrum (AS) with markers for Ladby C30238, L4 504, internal number A.


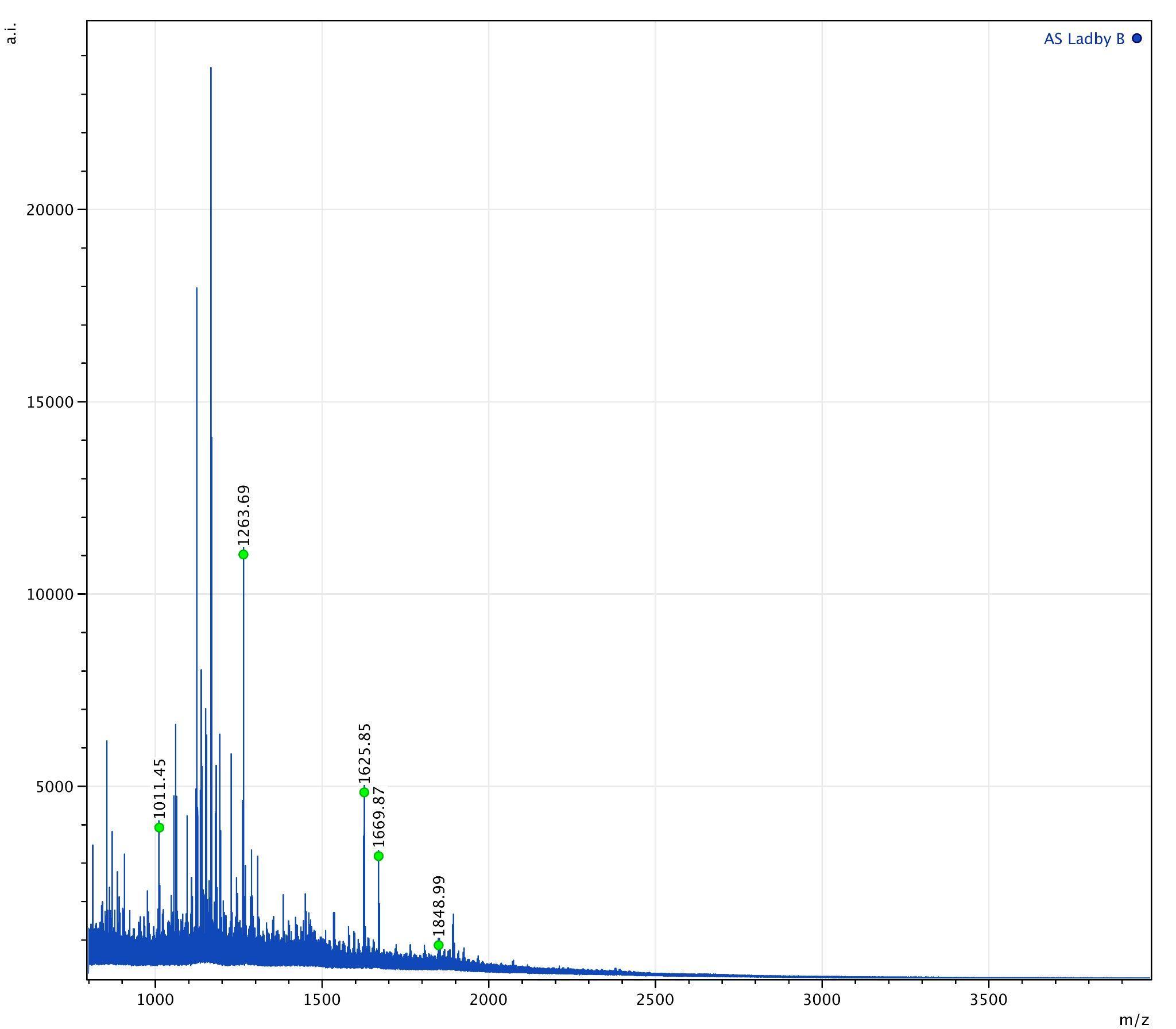


**Figure S14.** Averaged spectrum (AS) with markers for Ladby C30238, L4 504, internal number B.


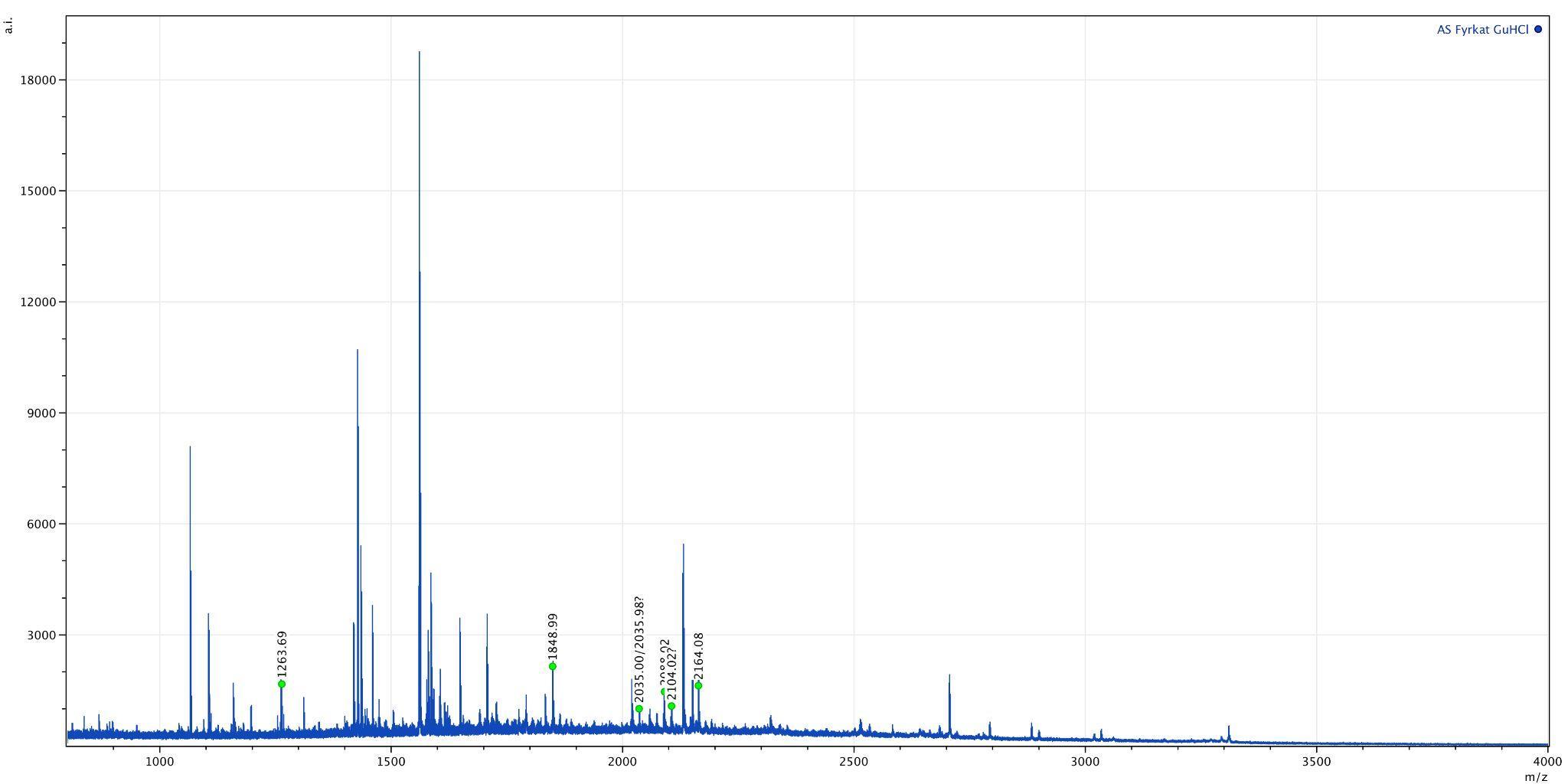


**Figure S15.** Averaged spectrum (AS) with markers for Fyrkat, Grave 4, D158-1966, GuHCl protocol.


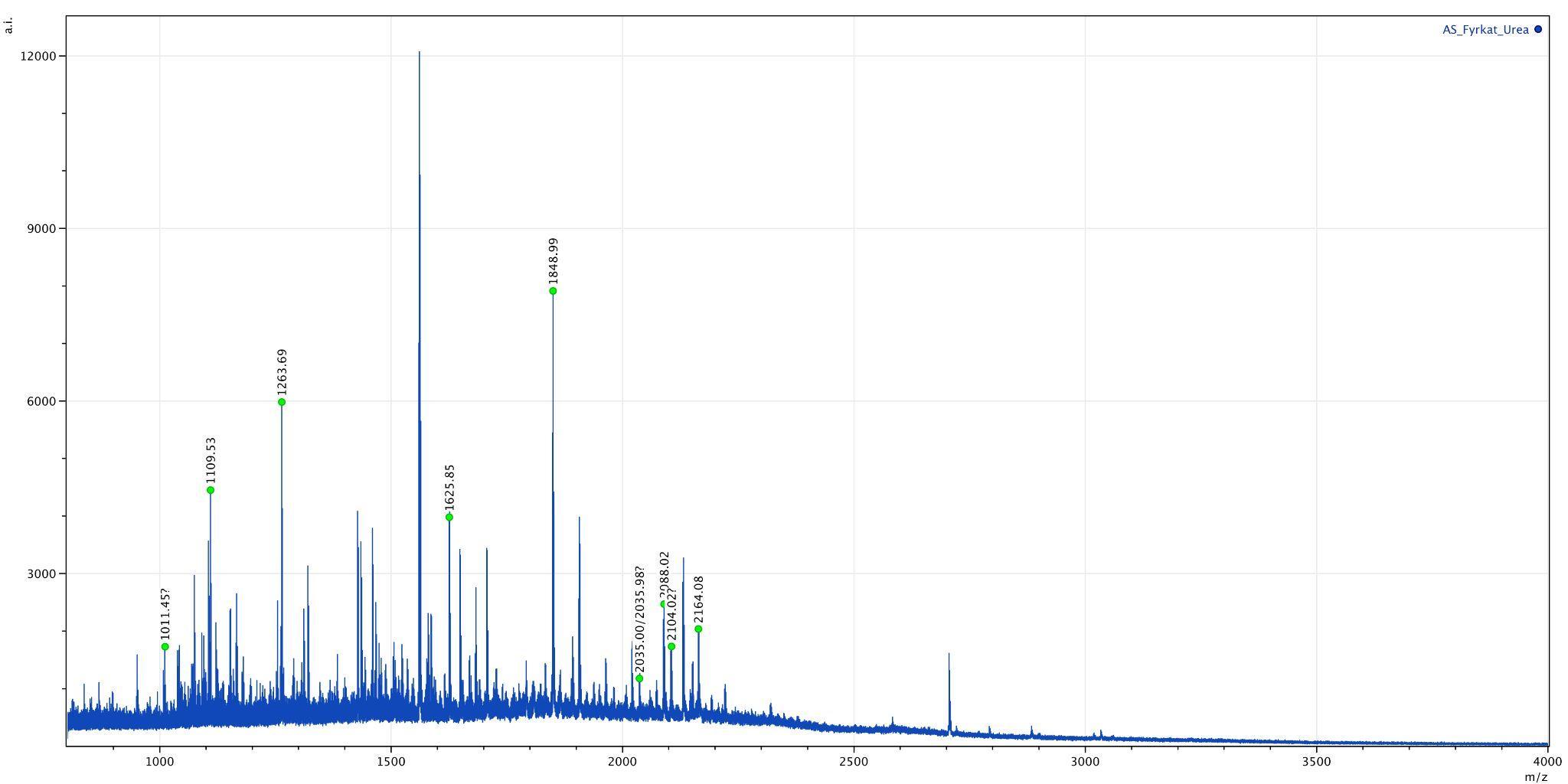


**Figure S16.** Averaged spectrum (AS) with markers for Fyrkat, Grave 4, D158-1966, Urea protocol.

**Text. LC-MS of fur samples**

Leftover peptide eluate for five samples (Hvilehøj C4273-97 fragment 1, 19 and 60, Bjerringhøj AdC143 and both Fyrkat extractions) was also used for LC-MS/MS analysis at the Novo Nordisk Foundation Center for Protein Research, the University of Copenhagen. The protein concentration of these subsamples was measured by Nanodrop (Thermo, Wilmington, DE, USA). Volumes equal to approximately 2 µg for each of the Fyrkat extractions and 1.2 µg for F1-3 and F8 samples were transferred to a 96 well plate and ACN was removed with a vacuum centrifuge. Samples were rehydrated in 10 μL (Fyrkat) or 12 µL of 0.1% TFA, 5% ACN. Five microliters per extraction/sample were then analysed on a 77 min gradient by an EASY-nLC 1200 (Proxeon, Odense, Denmark) coupled to either a Q Exactive HF-X (Thermo Scientific, Bremen, Germany) for the Fyrkat sample or an Orbitrap Exploris 480 (Thermo Scientific, Bremen, Germany) for all other samples. The LC-MS/MS parameters were the same as previously used for palaeoproteomic samples [[32]](https://paperpile.com/c/Tol2oT/7bPdM) or adapted from that for the Orbitrap Exploris 480. In short for the Q Exactive HF-X: MS1: 120k resolution, maximum injection time (IT) 25 ms, scan target 3E6. MS2: 60k resolution, top 10 mode, maximum IT 118 ms, minimum scan target 3E3, normalised collision energy of 28, dynamic exclusion 20 s, and isolation window of 1.2 m/z. For the Orbitrap Exploris 480, it was the same except for adjustments made for different machine settings, namely: MS1: AGC target of 300%. MS2: AGC target of 200% and HCD collision energy of 30.

**LC-MS/MS data analysis**

The Thermo RAW files were then searched using MaxQuant (v 1.6.3.4; [[33]](https://paperpile.com/c/Tol2oT/hqtrR)). Initial searches were against mammalian keratins downloaded (Feb-July 2021) from NCBI and Uniprot and subsequent searches eventually narrowed down to family or species based on the results of the ones before. The final databases were the results of keratin searches on NCBI and Uniprot for the following: *Castor* sp. (Hvilehøj C4273-97, fragment 19 and Bjerringhøj AdC143), *Ovis aries* (Hvilehøj C4273-97, fragment 1), Sciuridae (Hvilehøj C4273-97, fragment 60), and Mustelidae (Fyrkat). In the case of Hvilehøj C4273-97, fragment 60, it was noted that the local Sciuridae, red squirrel (*Sciurus vulgaris*), is not available in any public database. The closest relative with some keratin sequence data available is the grey squirrel (*Sciurus carolinensis,* native to North America) but, however, not all keratin sequences were available. Therefore, we decided to search the *S. vulgaris* genome (mSciVul1.2; GCA_902686455.2) for keratin proteins. This was done by a tblastn genome search using 17 known keratin CDS sequences of the Marmotini proteins already identified in the sample as a query. All hits with an e-value lower than 1e-5, similarity greater than 80% and less than 2 gap opens (135 in total) were considered as potential *S. vulgaris* fragment keratin sequences and added to the database. The combined fasta file with the publically available Sciuridae sequences and the potential *S. vulgaris* keratin fragments is uploaded to PRIDE with the raw data (PXD030529).
At the same time, samples were searched against the contaminant database provided by MaxQuant, containing common sources of laboratory protein contamination, including human keratins (from skin and hair, easily transferred to the sample by handling). Any contaminant proteins detected were not analysed further. The initial searches to narrow down the database were searched with a tryptic digestion, while the final databases were performed with a semi-tryptic search. Carbamidomethyl (C) was set as a fixed modification and variable modifications were oxidation (M), Acetyl (Protein N-term), Deamidation (NQ), Gln ->pyro-Glu, and Glu ->pyro-Glu. For all searches the false discovery rate (FDR) was at 0.01 (1%). All other settings were the software defaults, except that the minimum score for unmodified and modified peptides searches was set at 60.

Proteins were confidently assigned when at least two razor+unique peptides covering distinct areas of the sequence were identified. Proteins with solely completely overlapping peptides were discarded in the final results. In the final searches, all peptides were run through BLASTp (<https://blast.ncbi.nlm.nih.gov/Blast.cgi?PAGE=Proteins>, [[34]](https://paperpile.com/c/Tol2oT/w0VVV) to determine species specificity. This was also done in the earlier searches for the unique peptides to determine reliable identifications used to narrow down the database. In addition, MS/MS spectra of species specific peptides were manually inspected for further confidence in the identifications. A species specific peptide was not used for species identification if it did not meet manual inspection criteria (generally high intensity peaks, few unidentified peaks, and almost, if not all, b and y ions present). Full results of the final searches are available in Dataset S1.

In addition, deamidation was calculated using publicly available code [[32]](https://paperpile.com/c/Tol2oT/7bPdM), with contaminant peptides (based on razor protein) filtered out of the samples, and just based on contamination for the two Fyrkat blanks for control of laboratory-induced deamidation. Figure S17 shows the results of this calculation.

**Table S6.** Species identifications based on the LC-MS/MS with semi-tryptic searches. * Not many available sequences for *Castor fiber* (European beaver) so North American beaver is used as surrogate. † Due to location and age of the samples, the other mustelids in the database are unlikely, which would also agree with the GuHCl subsample. ‡ Sample numbers in PRIDE refer to these.

| **Site and inventory no.** | **Lab ID‡** | **Species ID** | **No. of Proteins** | **No. of Identified Razor Peptides** | **No. of MS/MS spectra** |
| --- | --- | --- | --- | --- | --- |
| Hvilehøj C4273-97, fragment 19 | F1 | *Castor* (*canadensis*)*** | 10 | 164 | 560 |
| Hvilehøj C4273-97, fragment 1 | F2 | *Ovis aries* | 12 | 341 | 1075 |
| Hvilehøj C427397, fragment 60 | F3 | Sciuridae, most likely *Sciurus vulgaris* | 17 | 279 | 1093 |
| Bjerringhøj, AdC143 | F8 | *Castor* (*canadensis*)*** | 11 | 226 | 972 |
| Fyrkat, Grave 4, D158-1966,  GuHCl protocol | Fyrkat GuHCl | *Mustela* sp. | 22 | 858 | 2339 |
| Fyrkat, Grave 4, D158-1966,  Urea protocol | Fyrkat Urea | Mustelidae (probably *Mustela* sp. †) | 20 | 286 | 847 |


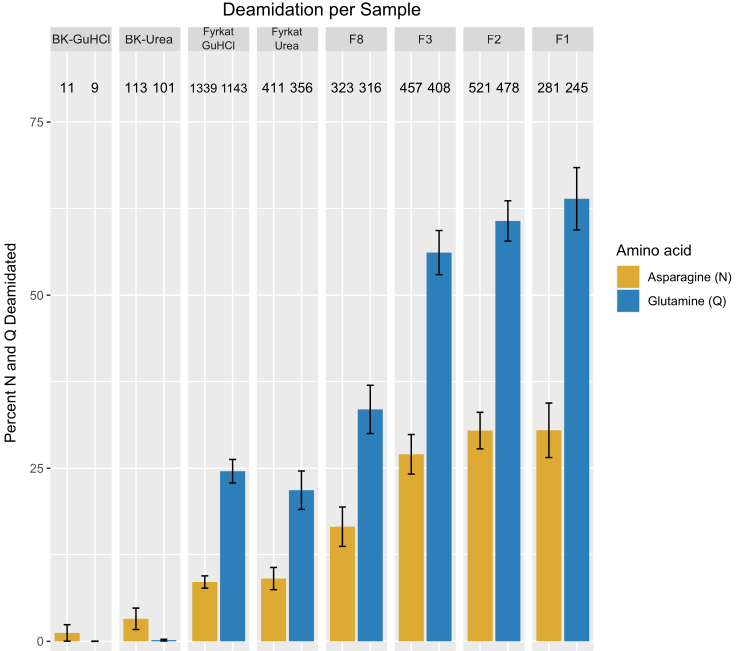


**Figure S17.** Deamidation levels of the LC-MS/MS samples and blanks: Overall percentage of deamidation for asparagine (N) and glutamine (Q) residues for peptides from the archaeological samples, excluding contaminants. The two blank samples are also included, containing only contamination. Error bars represent standard deviations and the numbers above each bar represent the numbers of peptides on which the calculations are based.

**Text. Microscopy of fur**

For microscopic analysis, two different mountings of fur were used for the identifications: longitudinal and cross-sectional.

A few hairs from each fur sample were taken, using sterile tweezers and disposable gloves. For the longitudinal mounting, a few hairs were degreased in acetone, and mounted with Pertex Mounting Medium ® on a slide, as parallel as possible to the slide edge and a coverslip was placed on top. Air bubbles were avoided by gently applying pressure to the coverslip. The mounted samples were left to dry for one day in a fume cupboard.

For the cross-section, a stack of hairs from each fur sample were selected. In order to keep the hairs as parallel as possible, they were tied together at both ends using cotton thread. The hair was then fixed in Glutaraldehyde in 0.05M Phosphate buffer. Subsequently, it was stained using 1% OsO_4_ and 0.05M (1.5%) K_3_FeCn_6_ before being washed in H_2_O. Hereafter, the fur specimens were dehydrated in an ethanol gradient, followed by infiltration with propylene oxide. Afterwards, the bundle of hair was embedded in a mould filled with epoxy resin. The embedded samples were polymerized at 60ºC for at least 24 hours. The EPON-blocks were sectioned to the area of interest with a microtome, Leica Ultracut UCT. Cross-sections (thickness 1 µm) were cut for transmission light microscopy, and stained with 1% toluidine blue and mounted with Pertex as described above. Cross-sectional preparations were carried out at the University of Copenhagen at the Core Facility for Integrated Microscopy (CFIM), in collaboration with Professor Klaus Qvortrup and Lab Technician Zhila Nikrozi.

Microscopy of longitudinally mounted hairs and cross-sections was performed with a Leica DM4 M using transmitted light (TLM) (100x, 200x, 400x, and 500x magnification).

The longitudinal specimens were described with regards to the presence of primary and secondary hairs. Mean measurements of total hair width were recorded. The cuticular patterns were described according to Wildman [[35]](https://paperpile.com/c/Tol2oT/Zmck/?noauthor=1) and Teerink [[36]](https://paperpile.com/c/Tol2oT/wSWzi/?noauthor=1) with regards to scale position, scale patterns, structure of scale margins and the distance between them. The composition of medulla was described with regards to structure and form, as well as measurement of width. Where pigmentation was present, it was noted. The cross-sectioned specimens were described according to Wildman and Teerink [[36,37]](https://paperpile.com/c/Tol2oT/uP4oa+wSWzi), regarding hair’s outer shape.

After compiling information from the longitudinal and cross-sectioned specimens, visual comparison was made with photos of available specimens from the Danish National Museum’s objects’ and reference collection, and appropriate reference literature (especially regarding European mammals) [[35–41]](https://paperpile.com/c/Tol2oT/i9jNw+hx8Gi+wSWzi+Zmck+JxCX6+uP4oa+MKn07). Species were assigned based on as many concurrent parameters as possible in order to provide the most valid identification. Results are shown in Table S7.


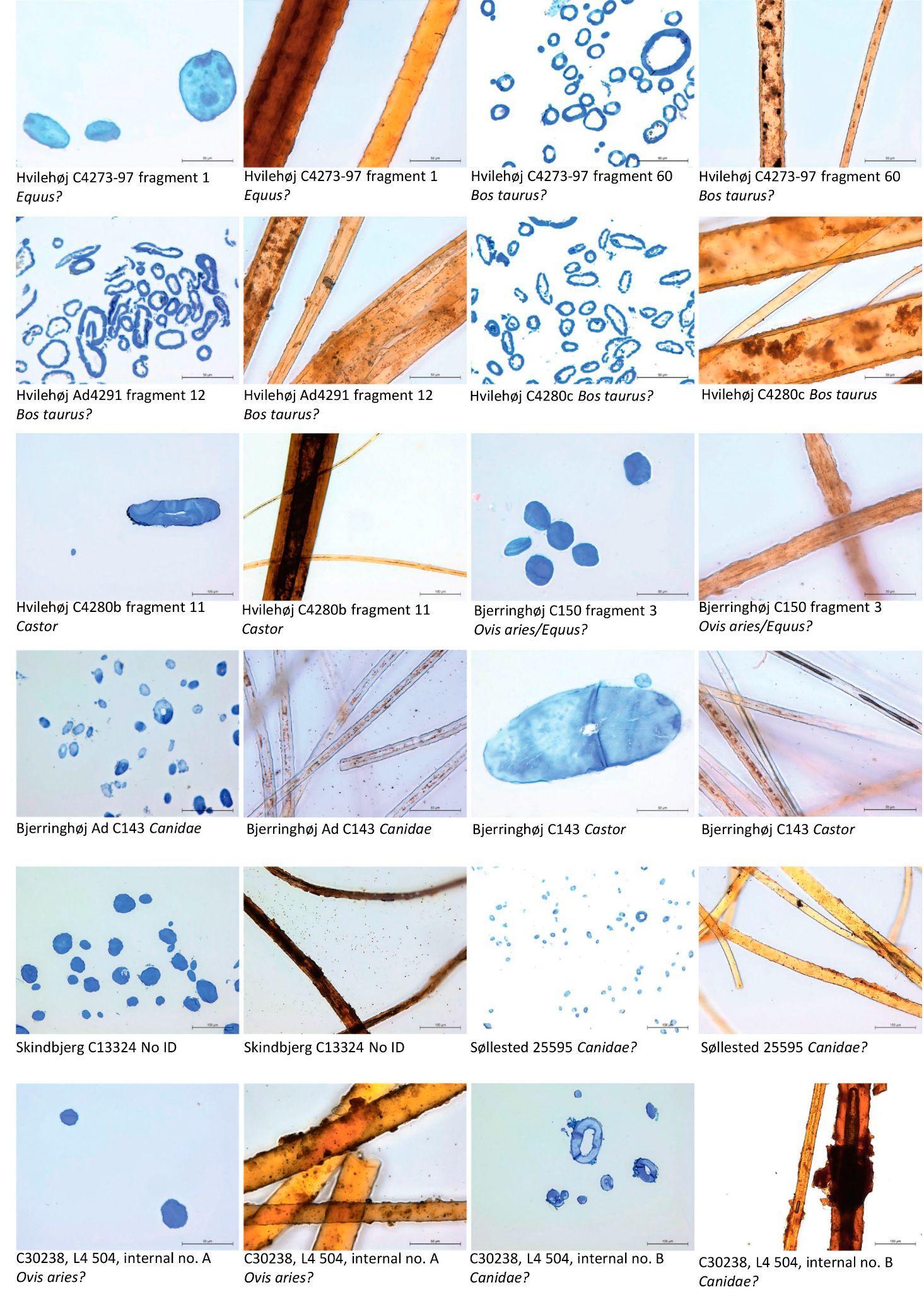


**Figure S18.** Hairs from all samples observed in cross-sections and longitudinally mounted. Photos: Anne Lisbeth Schmidt, the National Museum of Denmark.

**Table S7.** Overview of the identified species, based on cross-sections and longitudinally mounted primary and secondary hairs performed with transmitted light microscopy of hair by reference to literature [[35–40,42]](https://paperpile.com/c/Tol2oT/XdPZU+uP4oa+JxCX6+i9jNw+hx8Gi+wSWzi+Zmck).


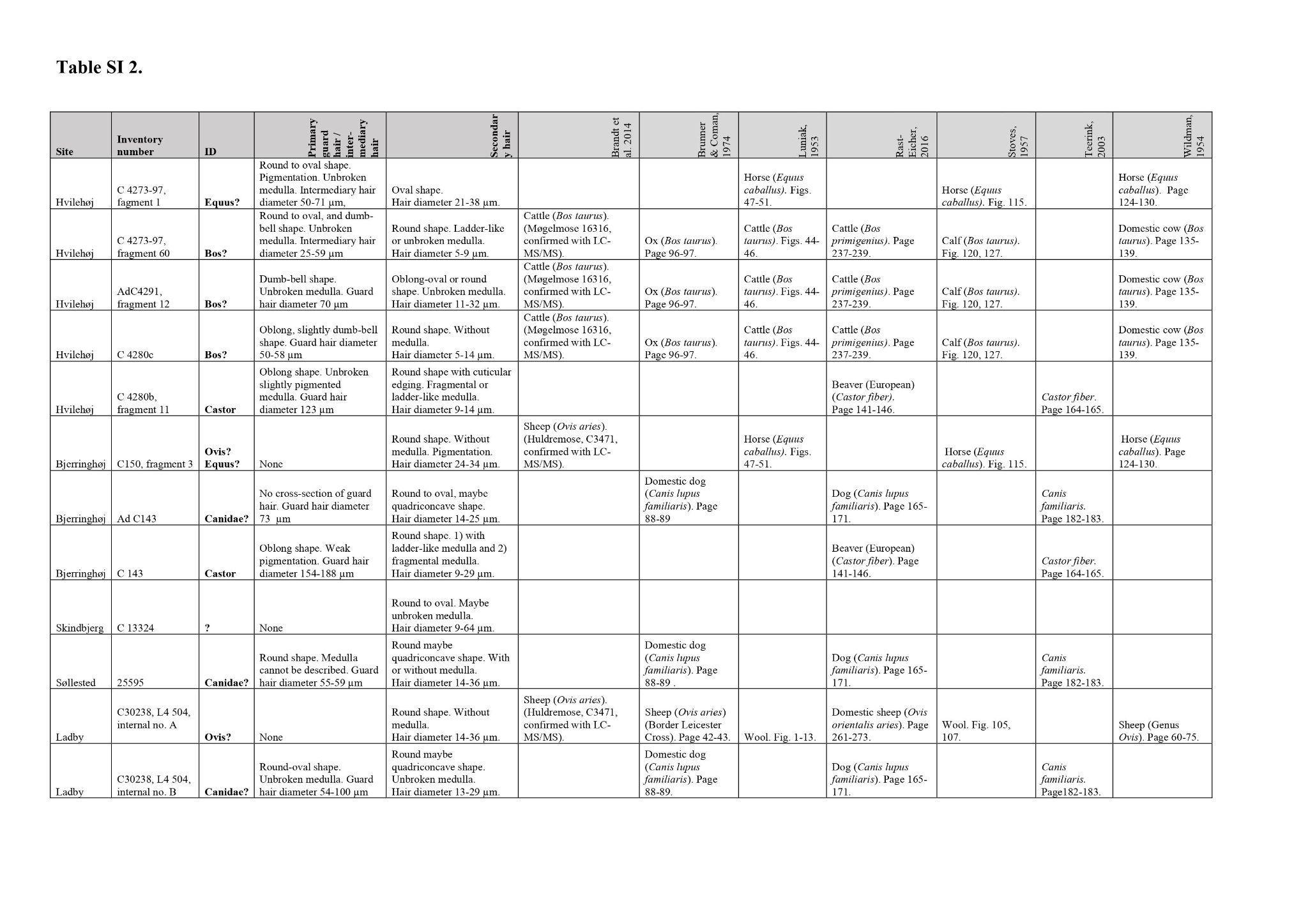


**SI References**

1. [Bender Jørgensen L. Forhistoriske textiler i Skandinavien. Prehistoric Scandinavian Textiles. Nordiske Fortidsminder. 1986.](http://paperpile.com/b/Tol2oT/g0im4)

2. [Krag AH, Knudsen LR. Vikingetidstekstiler. Nye opdagelser fra gravfundene i Hvilehøj og Hørning. Denmark Nationalmuseet Nationalmuseets Arbejdsmark. 1999; 159–170.](http://paperpile.com/b/Tol2oT/2kisa)

3. [Iversen M, Näsman U, Vellev J. Mammen: Grav, kunst og samfund i vikingetid. Moesgård; Jysk arkaeologisk selskab; Århus; I kommission hos Aarhus …; 1991.](http://paperpile.com/b/Tol2oT/JpVMf)

4. [Hald M. Olddanske tekstiler: komparative tekstil- og dragthistoriske studier paa grundlag af mosefund og gravfund fra jernalderen : with an English summary. I. kommission hos gyldendalske boghandel; 1950.](http://paperpile.com/b/Tol2oT/QDRKX)

5. [Worsaae JJA. Om Mammen-Fundet fra Hedenskabets Slutningstid. Thiele; 1869.](http://paperpile.com/b/Tol2oT/5LGFj)

6. [Brønsted J. Danish Inhumation Graves of the Viking Age: A Survey. Acta Archaeol. 1936;7: 81–228.](http://paperpile.com/b/Tol2oT/bpbp2)

7. [Pedersen A. Søllested-nye oplysninger om et velkendt fund. Aarbøger for nordisk Oldkyndighed og Historie. 1996.](http://paperpile.com/b/Tol2oT/ohLqM)

8. [Thorvildsen K. Ladby-skibet. Lynge i komm.; 1957.](http://paperpile.com/b/Tol2oT/Iakrc)

9. [Sørensen AC. Ladby: A Danish Ship-grave from the Viking Age. Viking Ship Museum; 2001.](http://paperpile.com/b/Tol2oT/BHfVv)

10. [Roesdahl E. Fyrkat: en jysk vikingeborg. 2. Oldsagerne og gravpladsen. Lynge; 1977.](http://paperpile.com/b/Tol2oT/XRvYZ)

11. [Karg S, Mannering U, Pentz P, Baastrup MP. Kong Haralds vølve. Nationalmuseets Arbejdsmark. 2009;2009: 215–232.](http://paperpile.com/b/Tol2oT/eKfhz)

12. [Cappellini E, Jensen LJ, Szklarczyk D, Ginolhac A, da Fonseca RAR, Stafford TW, et al. Proteomic analysis of a pleistocene mammoth femur reveals more than one hundred ancient bone proteins. J Proteome Res. 2011;11: 917–926.](http://paperpile.com/b/Tol2oT/Jf7bt)

13. [Orlando L, Ginolhac A, Raghavan M, Vilstrup J, Rasmussen M, Magnussen K, et al. True single-molecule DNA sequencing of a pleistocene horse bone. Genome Res. 2011;21: 1705–1719.](http://paperpile.com/b/Tol2oT/N6Z9b)

14. [Gilbert MTP, Tomsho LP, Rendulic S, Packard M, Drautz DI, Sher A, et al. Whole-genome shotgun sequencing of mitochondria from ancient hair shafts. Science. 2007;317: 1927–1930.](http://paperpile.com/b/Tol2oT/QTe1)

15. [Allentoft ME, Sikora M, Sjögren K-G, Rasmussen S, Rasmussen M, Stenderup J, et al. Population genomics of Bronze Age Eurasia. Nature. 2015;522: 167–172.](http://paperpile.com/b/Tol2oT/aAgDw)

16. [Dabney J, Knapp M, Glocke I, Gansauge M-T, Weihmann A, Nickel B, et al. Complete mitochondrial genome sequence of a Middle Pleistocene cave bear reconstructed from ultrashort DNA fragments. Proc Natl Acad Sci U S A. 2013;110: 15758–15763.](http://paperpile.com/b/Tol2oT/V89Ca)

17. [Carøe C, Gopalakrishnan S, Vinner L, Mak SST, Sinding M-HS, Samaniego JA, et al. Single-tube library preparation for degraded DNA. Methods Ecol Evol. 2017.](http://paperpile.com/b/Tol2oT/XO8VC)

18. [Mak SST, Gopalakrishnan S, Carøe C, Geng C, Liu S, Sinding M-HS, et al. Comparative performance of the BGISEQ-500 vs Illumina sequencing platforms for palaeogenomic sequencing. Gigascience. 2017.](http://paperpile.com/b/Tol2oT/CvZvx)

19. [Meyer M, Kircher M. Illumina sequencing library preparation for highly multiplexed target capture and sequencing. Cold Spring Harb Protoc. 2010;2010: db.prot5448.](http://paperpile.com/b/Tol2oT/pOAXF)

20. [Schubert M, Ermini L, Der Sarkissian C, Jónsson H, Ginolhac A, Schaefer R, et al. Characterization of ancient and modern genomes by SNP detection and phylogenomic and metagenomic analysis using PALEOMIX. Nat Protoc. 2014;9: 1056–1082.](http://paperpile.com/b/Tol2oT/TYYWX)

21. [Fadlan I. Ibn Fadlan and the Land of Darkness: Arab Travellers in the Far North. Penguin UK; 2012.](http://paperpile.com/b/Tol2oT/EuICH)

22. [Martin J. Treasure of the Land of Darkness: The Fur Trade and its Significance for Medieval Russia. Cambridge University Press; 1986.](http://paperpile.com/b/Tol2oT/xdKkS)

23. [Korneliussen TS, Albrechtsen A, Nielsen R. ANGSD: Analysis of Next Generation Sequencing Data. BMC Bioinformatics. 2014;15: 356.](http://paperpile.com/b/Tol2oT/VgF7l)

24. [Sinding M-HS, Vieira FG, Smith MH. Unmatched DNA preservation prove arctic hare and sheep wool in Norse Greenlandic textile from “The Farm Beneath the Sand.” Journal of Archaeological Science: Reports. 2017;14: 603–608.](http://paperpile.com/b/Tol2oT/gAW4V)

25. [Solazzo C, Rogers PW, Weber L, Beaubien HF, Wilson J, Collins M. Species identification by peptide mass fingerprinting (PMF) in fibre products preserved by association with copper-alloy artefacts. J Archaeol Sci. 2014;49: 524–535.](http://paperpile.com/b/Tol2oT/33KSS)

26. [Ebsen JA, Haase K, Larsen R, Sommer DVP, Brandt LØ. Identifying archaeological leather – discussing the potential of grain pattern analysis and zooarchaeology by mass spectrometry (ZooMS) through a case study involving medieval shoe parts from Denmark. J Cult Herit. 2019. doi:](http://paperpile.com/b/Tol2oT/fck3M)[10.1016/j.culher.2019.04.008](http://dx.doi.org/10.1016/j.culher.2019.04.008)

27. [Strohalm M, Hassman M, Kosata B, Kodícek M. mMass data miner: an open source alternative for mass spectrometric data analysis. Rapid Commun Mass Spectrom. 2008;22: 905–908.](http://paperpile.com/b/Tol2oT/2ORgY)

28. [Solazzo C, Wadsley M, Dyer JM, Clerens S, Collins MJ, Plowman J. Characterisation of novel α-keratin peptide markers for species identification in keratinous tissues using mass spectrometry. Rapid Commun Mass Spectrom. 2013;27: 2685–2698.](http://paperpile.com/b/Tol2oT/QhVNq)

29. [Solazzo C. Follow‐up on the characterization of peptidic markers in hair and fur for the identification of common North American species. Rapid Commun Mass Spectrom. 2017. Available:](http://paperpile.com/b/Tol2oT/UbrNz) <http://onlinelibrary.wiley.com/doi/10.1002/rcm.7923/full>

30. [Hollemeyer K, Altmeyer W, Heinzle E. Identification and quantification of feathers, down, and hair of avian and mammalian origin using matrix-assisted laser desorption/ionization time-of-flight mass spectrometry. Anal Chem. 2002;74: 5960–5968.](http://paperpile.com/b/Tol2oT/ktnJy)

31. [Solazzo C, Wadsley M, Dyer JM, Clerens S, Collins MJ, Plowman J. Characterisation of novel α-keratin peptide markers for species identification in keratinous tissues using mass spectrometry. Rapid Commun Mass Spectrom. 2013;27: 2685–2698.](http://paperpile.com/b/Tol2oT/CG7t)

32. [Mackie M, Rüther P, Samodova D, Di Gianvincenzo F, Granzotto C, Lyon D, et al. Palaeoproteomic Profiling of Conservation Layers on a 14th Century Italian Wall Painting. Angewandte Chemie International Edition. 2018;57: 7369–7374.](http://paperpile.com/b/Tol2oT/7bPdM)

33. [Cox J, Mann M. MaxQuant enables high peptide identification rates, individualized p.p.b.-range mass accuracies and proteome-wide protein quantification. Nature Biotechnology. 2008. pp. 1367–1372. doi:](http://paperpile.com/b/Tol2oT/hqtrR)[10.1038/nbt.1511](http://dx.doi.org/10.1038/nbt.1511)

34. [Altschul SF, Gish W, Miller W, Myers EW, Lipman DJ. Basic local alignment search tool. J Mol Biol. 1990;215: 403–410.](http://paperpile.com/b/Tol2oT/w0VVV)

35. [Wildman AB, Association WIR, Others. Microscopy of Animal Textile Fibres. 1954. Available:](http://paperpile.com/b/Tol2oT/Zmck) <https://agris.fao.org/agris-search/search.do?recordID=US201300380779>

36. [Teerink BJ. Hair of West European Mammals: Atlas and Identification Key. Cambridge University Press; 2004.](http://paperpile.com/b/Tol2oT/wSWzi)

37. [Brunner H. The identification of mammalian hair. Port Melbourne, VIC, Australia: Inkata Press; 1974.](http://paperpile.com/b/Tol2oT/uP4oa)

38. [Rast-Eicher A. Fibres: Microscopy of Archaeological Textiles and Furs. Archaeolingua Alapítvány; 2016.](http://paperpile.com/b/Tol2oT/i9jNw)

39. [Stoves JL. Fibre Microscopy: Its Technique and Application. National Trade Press; 1957.](http://paperpile.com/b/Tol2oT/hx8Gi)

40. [Luniak B. The Identification of Textile Fibres: Qualitative and Quantitative Analysis of Fibre Blends. Pitman; 1953.](http://paperpile.com/b/Tol2oT/JxCX6)

41. [Galatík, A. Galatík, J. Krul, Z. Galatík, A. Furskin Identification. 2011. Available:](http://paperpile.com/b/Tol2oT/MKn07) [www.furskin.cz](http://www.furskin.cz)

42. [Brandt LØ, Schmidt AL, Mannering U, Sarret M, Kelstrup CD, Olsen JV, et al. Species identification of archaeological skin objects from Danish bogs: comparison between mass spectrometry-based peptide sequencing and microscopy-based methods. PLoS One. 2014;9: e106875.](http://paperpile.com/b/Tol2oT/XdPZU)
